# A polycystin-2 protein with modified channel properties leads to an increased diameter of renal tubules and to renal cysts

**DOI:** 10.1242/jcs.259013

**Published:** 2021-08-23

**Authors:** Melanie Grosch, Katrin Brunner, Alexandr V. Ilyaskin, Michael Schober, Tobias Staudner, Denise Schmied, Tina Stumpp, Kerstin N. Schmidt, M. Gregor Madej, Thaissa D. Pessoa, Helga Othmen, Marion Kubitza, Larissa Osten, Uwe de Vries, Magdalena M. Mair, Stefan Somlo, Markus Moser, Karl Kunzelmann, Christine Ziegler, Silke Haerteis, Christoph Korbmacher, Ralph Witzgall

**Affiliations:** 1 Institute for Molecular and Cellular Anatomy, University of Regensburg, 93053 Regensburg, Germany; 2 Institute of Cellular and Molecular Physiology, Friedrich-Alexander University of Erlangen-Nürnberg, 91054 Erlangen, Germany; 3 Department of Biophysics, University of Regensburg, 93053 Regensburg, Germany; 4 Faculty of Biology and Preclinical Medicine, University of Regensburg, 93053 Regensburg, Germany; 5 Departments of Medicine and Genetics, Yale University, New Haven, CT 06520, USA; 6 Institute of Experimental Hematology, Technical University of Munich, 81675 Munich, Germany; 7 Department of Physiology, University of Regensburg, 93053 Regensburg, Germany

**Keywords:** Polycystin-2, PKD2, Autosomal-dominant polycystic kidney disease, Knock-in mice, Electrophysiology, *Xenopus laevis* oocytes, Tubular diameter, Lumen formation

## Abstract

Mutations in the *PKD2* gene cause autosomal-dominant polycystic kidney disease but the physiological role of polycystin-2, the protein product of *PKD2*, remains elusive. Polycystin-2 belongs to the transient receptor potential (TRP) family of non-selective cation channels. To test the hypothesis that altered ion channel properties of polycystin-2 compromise its putative role in a control circuit controlling lumen formation of renal tubular structures, we generated a mouse model in which we exchanged the pore loop of polycystin-2 with that of the closely related cation channel polycystin-2L1 (encoded by *PKD2L1*), thereby creating the protein polycystin-2^poreL1^. Functional characterization of this mutant channel in *Xenopus laevis* oocytes demonstrated that its electrophysiological properties differed from those of polycystin-2 and instead resembled the properties of polycystin-2L1, in particular regarding its permeability for Ca^2+^ ions. Homology modeling of the ion translocation pathway of polycystin-2^poreL1^ argues for a wider pore in polycystin-2^poreL1^ than in polycystin-2. In *Pkd2*^poreL1^ knock-in mice in which the endogenous polycystin-2 protein was replaced by polycystin-2^poreL1^ the diameter of collecting ducts was increased and collecting duct cysts developed in a strain-dependent fashion.

## Abstract (precis)

**Summary:** Replacement of the pore region of polycystin-2 with that of polycystin-2L1 results in wider renal tubules and polycystic kidney disease, thus demonstrating the essential function of its ion channel properties.

## INTRODUCTION

Mutations in the *PKD1* and *PKD2* genes lead to autosomal-dominant polycystic kidney disease (ADPKD), and patients suffering from this disease develop kidney cysts throughout their life. Human polycystin-2, the protein product of the *PKD2* gene, belongs to the transient receptor potential (TRP) family of non-selective cation channels. In previous experiments, it was shown that polycystin-2 conducts Ca^2+^ ions (Hanaoka et al., 2000) although K^+^ and Na^+^ ions are much preferred over Ca^2+^ ions (Liu et al., 2018; Shen et al., 2016). Crucial new insight into the molecular function of polycystin-2 has come from its structural characterization. It was shown by cryo-electron microscopy that polycystin-2 forms homo-tetrameric channels (Grieben et al., 2017; Shen et al., 2016; Wilkes et al., 2017). The pore-forming domain lies between the fifth and sixth membrane-spanning segment and is covered by a large domain connecting the first and second membrane-spanning segment. This domain is alternatively called the polycystin domain (Shen et al., 2016) or the tetragonal opening for polycystin (TOP) domain (Grieben et al., 2017), and is believed to be essential for regulating the channel properties of polycystin-2.

Like polycystin-2, polycystin-2L1 belongs to the TRPP branch of transient receptor potential channels. Although no mutations in the *PKD2L1* gene have been identified in patients suffering from polycystic kidney disease to date and *Pkd2l1* knock-out mice do not develop kidney cysts (Delling et al., 2013), polycystin-2L1 has attracted particular attention because it has been shown to act as a Ca^2+^ channel in primary cilia (DeCaen et al., 2013; Delling et al., 2013). Its molecular structure resembles that of polycystin-2 because polycystin-2L1 is also assembled as a tetramer and it also contains a polycystin/TOP domain (Hulse et al., 2018; Su et al., 2018b; Zheng et al., 2018). However, the channel opening of polycystin-2L1 is larger than that of polycystin-2, thus explaining its Ca^2+^ conductance.

The consequences resulting from the various types of mutations in the *PKD2* gene are poorly understood. Nonsense mutations, as well as many insertions, deletions and splice mutations, are predicted to lead to the loss of large portions of the polycystin-2 protein; accordingly they reveal little about the importance of individual regions of the protein. In contrast, missense mutations have the potential to yield valuable insight into the function of distinct domains and even single amino acids. For example, several likely and highly likely pathogenic missense mutations have been identified in the pore loop of polycystin-2 (https://pkd.mayo.edu/), thus strongly suggesting that the channel properties are essential for polycystin-2 to fulfil its physiological function. To test the hypothesis that altering the pore domain of polycystin-2 is sufficient to cause renal cyst formation, we have created a *Pkd2* knock-in mouse model, *Pkd2*^poreL1^, in which the mutated polycystin-2 protein contains the pore region of polycystin-2L1. The mutant protein is still active as a cation channel, although with different electrophysiological properties; specifically the mutant protein conducts Ca^2+^ ions more readily than wild-type polycystin-2. *Pkd2*^poreL1^ knock-in mice present with enlarged collecting ducts and develop renal cysts in a strain-dependent fashion. Our data reveal (1) that the ion channel properties are essential for polycystin-2 to exert its regular function, (2) that mutations affecting the ion channel properties of polycystin-2 are sufficient to cause ADPKD, and (3) that the genetic background plays a role in determining the severity of the disease.

## RESULTS

### Characterization of a polycystin-2^poreL1^ mutant protein in the *Xenopus laevis* oocyte expression system

**Fig. 1.**
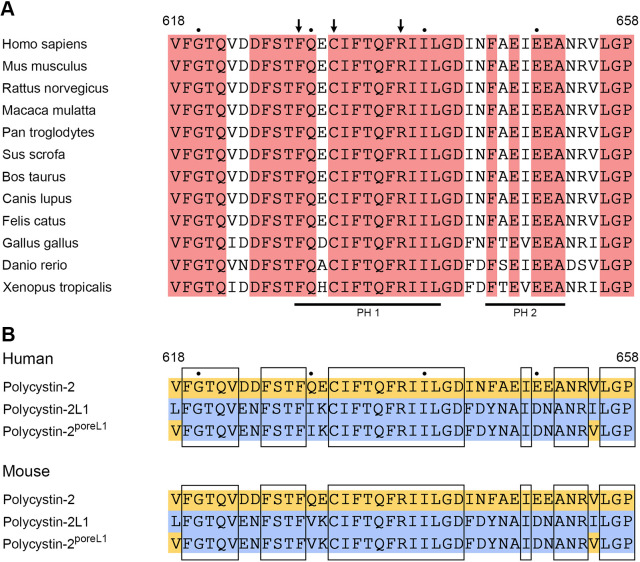
**Sequence comparison.** (A) Sequence comparison of the polycystin-2 pore region from different species. Identical residues are highlighted in red, the numbers above the sequence correspond to the residues in the human polycystin-2 protein. The bars below the sequence indicate pore helix 1 (PH 1, amino acids F629–L641) and 2 (PH 2, amino acids F646–A652). Arrows point to the amino acids affected by likely and highly likely pathogenic missense mutations, i.e. F629S, C632R, R638C (https://pkd.mayo.edu/). (B) Sequence comparisons of the pore region from polycystin-2 (yellow), polycystin-2L1 (blue) and polycystin-2^poreL1^. Identical residues between polycystin-2 and polycystin-2L1 are boxed.

The pore-forming loop of polycystin-2 is highly conserved in different species (Fig. 1A). In order to alter the ion channel properties of polycystin-2 we replaced its pore-forming loop with that of the closely related protein polycystin-2L1, thus creating the polycystin-2^poreL1^ mutant protein (Fig. 1B). The usefulness of such a pore-swapping strategy has previously been demonstrated by another group, which used the opposite approach and transferred the pore region of polycystin-2 to polycystin-2L1 (Shen et al., 2016).

**Fig. 2.**
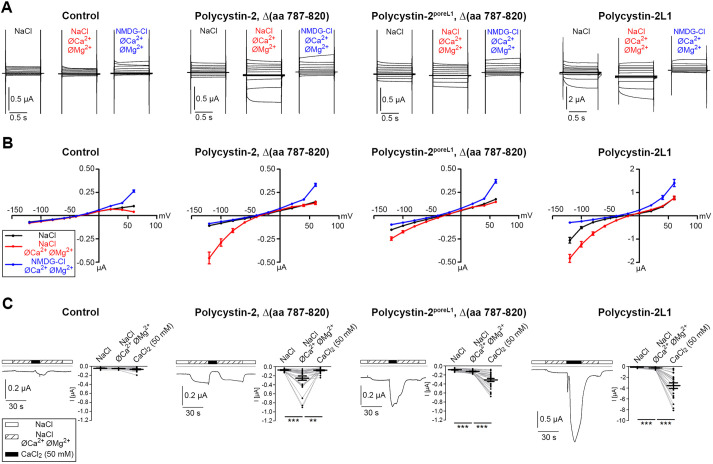
**Characterization of polycystin-2^poreL1^ in *Xenopus* oocytes.***Xenopus laevis* oocytes were injected with cRNAs encoding polycystin-2, polycystin-2^poreL1^ and polycystin-2L1. In the case of polycystin-2 and polycystin-2^poreL1^ the 34-amino-acid domain responsible for the preferential location of the respective protein in the endoplasmic reticulum was deleted to achieve consistent incorporation into the plasma membrane. (A) The change from a standard bath solution (NaCl) to a solution without divalent cations (NaCl, øCa^2+^, øMg^2+^) had no effect on control oocytes but stimulated Na^+^ inward currents in oocytes expressing polycystin-2, Δ(aa 787–820), polycystin-2^poreL1^, Δ(aa 787–820) and polycystin-2L1, although to different degrees. Subsequent replacement of NaCl by NMDG-Cl abolished Na^+^ inward currents (NMDG-Cl, øCa^2+^, øMg^2+^). For each condition, representative overlays of ten individual whole-cell current traces are shown which were obtained from consecutive 1-s voltage steps in 20 mV increments starting with a hyperpolarizing pulse to −120 mV from a holding potential of −60 mV. (B) Current data of the final 300 ms of the pulses were taken from similar experiments to construct corresponding average *I*/*V* curves. Results represent experiments from 28–36 oocytes and five different oocyte preparations, shown are mean±s.e.m. (C) In another set of experiments, oocytes were sequentially exposed to the standard bath solution (NaCl, open bar), a NaCl bath solution without divalent cations (NaCl, øCa^2+^, øMg^2+^, hatched bar) and a bath solution containing 50 mM CaCl_2_ (CaCl_2_, filled bar) as indicated. Representative whole-cell current traces recorded at a continuous holding potential of −80 mV (left panels) demonstrate that exposure to 50 mM CaCl_2_ elicits large inward current responses in oocytes expressing polycystin–2^poreL1^ and polycystin-2L1 (the dotted line indicates zero current levels). In contrast, addition of 50 mM CaCl_2_ reversibly inhibits an inward current component in polycystin-2-expressing oocytes. Whole-cell currents in control oocytes are largely unaffected by 50 mM CaCl_2_. The summary graphs (right panels) show the plateau inward currents in the standard bath solution and in a bath solution lacking divalent cations as well as the maximum inward currents reached in the presence of 50 mM CaCl_2_. 21–26 oocytes from three different oocyte preparations were used per experimental group. Measurements from individual oocytes and the mean±s.e.m. are shown. **P<0.01, ***P<0.001 (paired two-tailed *t*-test).

The electrophysiological characterization of polycystin-2 channel properties has been hampered by the limited integration of the channel into the plasma membrane. This is due, at least in part, to a 34-amino-acid domain located in the C-terminus of polycystin-2 (Cai et al., 1999; Hoffmeister et al., 2011). Therefore, we generated polycystin-2 and polycystin-2^poreL1^ constructs lacking this motif to study the electrophysiological effects of replacing the pore region of polycystin-2 with that of polycystin-2L1. Deletion of the 34-amino-acid domain led to an increased expression of polycystin-2 at the cell surface in *Xenopus laevis* oocytes and facilitated the detection of polycystin-2-mediated currents (Fig. S1). For comparison, we investigated polycystin-2L1, which is known to express well at the plasma membrane with easily detectable currents (Chen et al., 1999; Li et al., 2002; Zheng et al., 2016). It has been reported that polycystin-2 with a gain-of-function mutation is strongly inhibited by Ca^2+^ and Mg^2+^ ions on the outside (Arif Pavel et al., 2016). Therefore, we hypothesized that we could elicit polycystin-2-mediated inward Na^+^ currents by removing divalent cations from the bath solution. In control oocytes, the simultaneous removal of Ca^2+^ and Mg^2+^ ions and subsequent replacement of Na^+^ by the large organic cation NMDG^+^ had no noticeable effect on the magnitude of baseline inward currents. This indicates that the endogenous Na^+^ conductance of control oocytes is negligible in the presence and absence of divalent cations. In contrast, the simultaneous removal of Ca^2+^ and Mg^2+^ ions resulted in a substantial inward current component in oocytes expressing polycystin-2 (Fig. 2A,B; Fig. S1). This inward current component was completely abolished by the subsequent replacement of Na^+^ by NMDG^+^, which demonstrated that it was carried by Na^+^. Importantly, the inward currents measured with NMDG^+^ in the absence of divalent cations were similar to those measured in the presence of Na^+^ and divalent cations. Thus, polycystin-2-mediated inward Na^+^ currents are almost completely inhibited by divalent cations, but can be stimulated by the simultaneous removal of Ca^2+^ and Mg^2+^ ions from the bath solution. In oocytes expressing polycystin-2^poreL1^ and polycystin-2L1, removal of Ca^2+^ and Mg^2+^ ions from the bath solution also activated an inward current component (Fig. 2A,B). In general, polycystin-2L1-mediated currents were larger than polycystin-2- and polycystin-2^poreL1^-mediated currents, possibly due to the higher density of channels at the cell surface. Using a similar experimental design as shown in Fig. 2A,B, we also tested the effect of replacing Na^+^ by K^+^ and Li^+^ on the inward currents mediated by polycystin-2, polycystin-2^poreL1^ and polycystin-2L1 in the absence of divalent cations (Fig. S2, Table S1). Our findings indicate that polycystin-2 conducts K^+^ considerably better than Na^+^ and Li^+^. In contrast, polycystin-2L1 conducts K^+^ and Na^+^ equally well but has a slightly reduced conductance for Li^+^. Importantly, the inward currents observed in oocytes expressing polycystin-2^poreL1^ showed intermediate properties.

We also investigated whether the Ca^2+^ permeability of polycystin-2^poreL1^ differed from that of polycystin-2. It has been shown that large transient inward currents can be elicited when cells expressing polycystin-2L1 are exposed to high extracellular Ca^2+^ concentrations (5–100 mM) (Chen et al., 1999; Li et al., 2002; Su et al., 2018b; Zheng et al., 2016). We confirmed these findings and observed a large transient inward current response in oocytes expressing polycystin-2L1 when they were exposed to a bath solution containing 50 mM CaCl_2_ (Fig. 2C). This response was not observed in control oocytes (Fig. 2C) and was not mimicked upon use of 50 mM MgCl_2_ (Fig. S3A). We therefore conclude that the stimulatory response required the expression of polycystin-2L1 and was Ca^2+^ dependent. The response was abolished in oocytes injected with the Ca^2+^ chelator EGTA (Fig. S3B) and in the presence of the chloride channel blocker niflumic acid (Fig. S3C). This indicates that the inward current response elicited by 50 mM CaCl_2_ is due to an activation of Ca^2+^-sensitive Cl^−^ channels by Ca^2+^ ions entering the cell through polycystin-2L1. Importantly, a similar transient inward current response was observed in oocytes expressing polycystin-2^poreL1^ upon changing to a bath solution containing 50 mM CaCl_2_ (Fig. 2C). In contrast, in oocytes expressing polycystin-2 switching to 50 mM CaCl_2_ caused no stimulatory response but reversibly inhibited an inward current component (Fig. 2C), consistent with the inhibition of polycystin-2 by divalent cations from the outside. Cell surface and intracellular expression of polycystin-2^poreL1^ appeared to be slightly lower than that of polycystin-2 (Fig. S4). Therefore the absence of an inward current response to 50 mM CaCl_2_ cannot be attributed to a reduced expression of polycystin-2 at the cell surface. These findings provide indirect evidence for a substantial Ca^2+^ permeability in oocytes expressing polycystin-2L1 and polycystin-2^poreL1^, which was not detectable in oocytes expressing polycystin-2 under the experimental conditions used. In summary, our functional studies in the oocyte expression system demonstrated that the pore properties of polycystin-2^poreL1^ differed from those of polycystin-2 and resembled those of polycystin-2L1, in particular regarding its Ca^2+^ permeability.

### Structural model

**Fig. 3.**
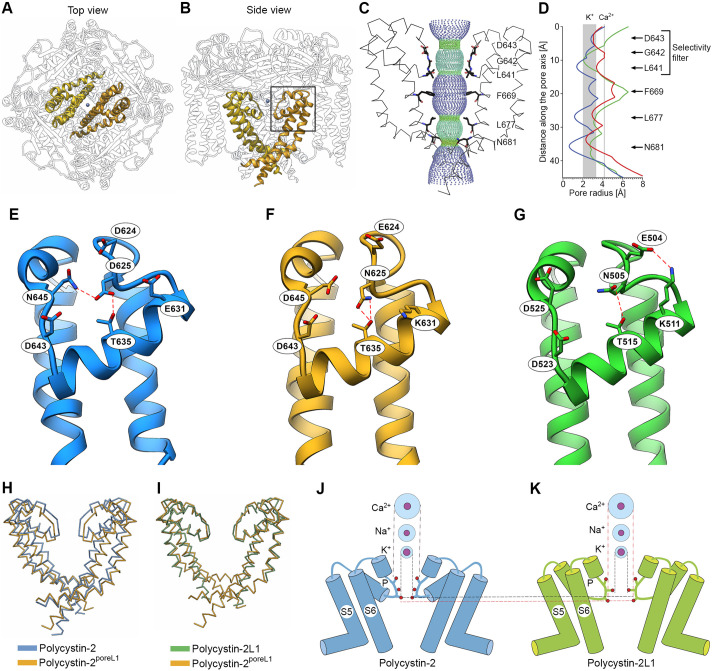
**Structural modeling of polycystin-2^poreL1^.** (A,B) Top view and side view of polycystin-2^poreL1^. The pore regions (amino acids 582–695) of two opposing protomers are highlighted in yellow and orange, the position of a cation which was observed in the original structure of wild-type polycystin-2 is indicated by the blue sphere. The rectangle indicates the region shown in more detail in panels E–G. (C) Profile of the ion-conduction pathway (dotted surface) shown along with two diagonally opposed protomers (wire diagrams) in polycystin-2^poreL1^. The amino acid residues in the selectivity filter and at the pore constrictions are depicted as sticks. (D) The pore radius is plotted along the ion-conduction axis together with the residues lining the pore. It can be seen that the most prominent constrictions are present in wild-type polycystin-2 (blue line) whereas the pore is wider in polycystin-2L1 (green line). The structure of polycystin-2^poreL1^ (red line) is closer to that of polycystin-2L1. Shaded regions indicate the radius of hydrated K^+^ and of hydrated Ca^2+^ ions (cf. Table S3). (E–G) Detailed view of the pore domain of polycystin-2, polycystin-2^poreL1^ and polycystin-2L1. In the case of polycystin-2, D625 mediates the interaction between T635 in pore helix 1 and N645 immediately adjacent to the N-terminal end of pore helix 2 (E). Such an arrangement is not observed in polycystin-2L1, where the corresponding N505 residue is located too far away to be able to interact with D525 although it can still interact with T515 (G). Since the pore domain in polycystin-2^poreL1^ contains those residues of polycystin-2L1 that are crucial for the tertiary structure just described, it is more similar to that of polycystin-2L1 than to that of polycystin-2 (F). (H,I) Superpositions of the pore domains of polycystin-2^poreL1^ (orange) and wild-type polycystin-2 (blue) (H), and of polycystin-2^poreL1^ (orange) and polycystin-2L1 (green) (I). The opening is wider in polycystin-2^poreL1^ than in polycystin-2, whereas the openings in polycystin-2^poreL1^ and polycystin-2L1 are very similar. (J,K) Diagram of the pore regions of polycystin-2 and polycystin-2L1. Shown are transmembrane segments S5 and S6 together with the intervening pore loop (P). The red circles indicate the amino acids in the selectivity filter of polycystin-2 (D643, G642 and L641, top to bottom) and of polycystin-2L1 (D523, G522 and L521, top to bottom). The outer circles of each ion indicate the radii of the hydrated ions, inner circles represent the ionic radii (cf. Table S3). It can be appreciated that hydrated K^+^ ions can pass through the selectivity filter of either pore loop but Ca^2+^ ions will only be able to pass through the selectivity filter of polycystin-2L1.

In order to better understand the decreased Ca^2+^ response observed for polycystin-2^poreL1^, the recently determined cryo-electron microscopical structures of full-length polycystin-2 (Wilkes et al., 2017) and polycystin-2L1 (Hulse et al., 2018; Su et al., 2018b) were used to model the effect of the 11 amino acid changes in polycystin-2^poreL1^. The pore domain in the wild-type polycystin-2 protein consists of two helices, pore helix 1 and 2, which are connected by an unfolded loop (Fig. 1A). This region contains several negatively charged and polar residues, which interact with each other and contribute to the stabilization of the pore architecture (Fig. 3A–G). Importantly, the end of pore helix 1 and the beginning of the loop between pore helices 1 and 2 is characterized by the presence of 3 amino acids (L641, G642 and D643) that constitute the selectivity filter of polycystin-2. Residue D643, whose orientation is crucial for the coordination and dehydration of cations entering the pore, is located at the entrance of the cation translocation pore together with the residue N645, which is coordinated by polar and charged residues of pore helix 1 (Fig. 3C–E).

The structure of polycystin-2L1 reveals several pronounced differences to the structure of polycystin-2. In the case of polycystin-2, amino acid D625 connects pore helices 1 and 2 by bridging T635 and N645 through its carboxyl side chain (Fig. 3E). Such an interaction for the corresponding amino acid, N505, is not observed in polycystin-2L1. Rather, loop 5, which lies between transmembrane segment 5 and pore helix 1, is oriented towards the N-terminal end of pore helix 1 by the potential interaction of E504 with K511 (Fig. 3G). The switch in the interaction network results in the lowering of pore helices 1 and 2 (Fig. 3H,I) and in the larger radius of the selectivity filter with respect to polycystin-2 (Fig. 3J,K). The structural model of polycystin-2^poreL1^ inherits the architecture of this region from polycystin-2L1 since all the involved sidechains have been introduced from polycystin-2L1 (Fig. 3F). However, although all 11 mutations are located in the pore region, the gate in membrane segment 6 (L677) is also open (Fig. 3C,D). The structural modeling supports a wider pore diameter in polycystin-2^poreL1^ with respect to polycystin-2 (Fig. 3J,K), and therefore emphasizes the experimental observations obtained in *Xenopus laevis* oocytes that polycystin-2^poreL1^ is permeable for the larger cation Ca^2+^ and polycystin-2 is not.

### Significance of the polycystin-2^poreL1^ protein for determining the diameter of renal tubules

**Fig. 4.**
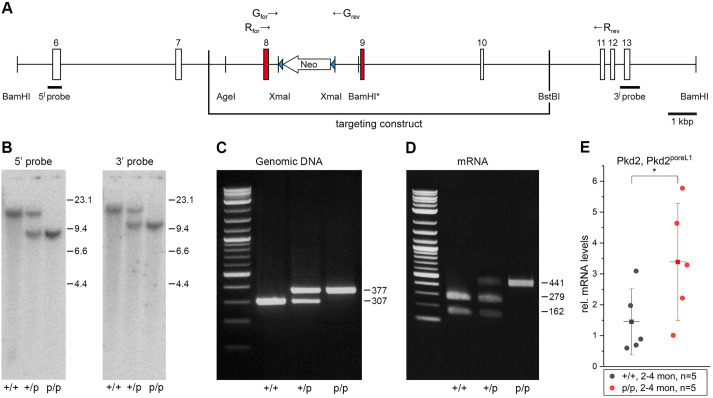
**Creation of *Pkd2*^poreL1^ knock-in mice.** (A) Targeting construct for the transfection of embryonic stem cells. Exons 8 and 9 contain the desired mutations (indicated by the red bars); the neomycin resistance gene flanked by loxP sites (blue triangles) was introduced into intron 8. Numbers above the bars indicate exons, G_for_ and G_rev_ indicate the position of the primers for PCR reactions from genomic DNA, and R_for_ and R_rev_ indicate the position of the primers for PCR reactions from mRNA. The BamHI site marked with an asterisk was introduced together with the nucleotides for the required amino acids. (B) Genomic DNA isolated from tail cuts of wild-type (+/+), heterozygous *Pkd2*^+/poreL1^ (+/p) and homozygous *Pkd2*^poreL1/poreL1^ (p/p) mice was digested with BamHI and hybridized with the respective 5′ and 3′ probes after Southern blotting. The presence of the expected bands can be seen in either case. Numbers indicate the respective sizes (in kb pairs) of the molecular mass standard. (C) PCR from genomic DNA (after Cre-mediated removal of the neomycin resistance gene) isolated from tail cuts run with the primers G_for_ and G_rev_. The mutated allele can be identified by its lower mobility; expected sizes are given on the right. (D) RT-PCR from total kidney RNA (again following Cre-mediated removal of the neomycin resistance gene) isolated from mice with the three different genotypes, after the PCRs, the products were digested with EcoRV. The PCR product of the wild-type allele can be digested with EcoRV whereas that of the mutated allele cannot. Expected sizes (in bp) are given on the right. (E) Quantitative PCR analysis of total RNA isolated from collecting ducts demonstrates higher mRNA levels for *Pkd2^poreL1^* than for *Pkd2*. Approximately 150 to 200 collecting ducts each were harvested from five mice at an age of 2 to 4 months. Shown are the mean±s.d. *P<0.05 (unpaired one-tailed *t*-test).

The data presented above provide strong evidence that the polycystin-2^poreL1^ protein conducts Ca^2+^ ions whereas the wild-type polycystin-2 protein does not. In order to test the hypothesis that the ion currents mediated by polycystin-2 play a key role for determining the width of renal tubules, we generated embryonic stem cells in which the exons of the endogenous *Pkd2* gene encoding the pore region were mutated so that a polycystin-2^poreL1^ protein would be generated (Fig. 4A). Successful germline transmission of the mutated embryonic stem cells was confirmed in heterozygous and homozygous *Pkd2* knock-in (*Pkd2*^poreL1^) mice by Southern blotting and PCR with genomic DNA (Fig. 4B,C). To confirm the expression of the wild-type and mutant mRNA, RT-PCR reactions were carried out with total RNA preparations from adult kidneys. Again the expected bands were seen (Fig. 4D), and sequencing of the PCR products confirmed the identity of the desired mutations (data not shown). Furthermore, just as for wild-type polycystin-2, the polycystin-2^poreL1^ mutant protein was detected in primary cilia and in the endoplasmic reticulum (Fig. S5). Having confirmed that our strategy was successful, heterozygous *Pkd2*^poreL1^ mice were backcrossed both onto a C57Bl/6 and a 129/Sv background for eight generations. This clean genetic background also allowed us to make sure that the introduction of the mutations had no negative effect on the transcription of the *Pkd2* gene. In light of the fact that our subsequent analysis (see below) focused on collecting ducts, we isolated collecting ducts from wild-type and homozygous *Pkd2* knock-in mice and subjected them to quantitative (q)PCR analysis. It turned out that the mRNA levels for polycystin-2^poreL1^ in collecting ducts from knock-in mice were somewhat higher than the *Pkd2* mRNA levels in wild-type mice (Fig. 4E). Our experience with the backcrosses had shown that the heterozygous knock-in mice were viable and their life expectancy was normal. To find out whether homozygous knock-in mice presented with a phenotype, *Pkd2*^+/poreL1^ knock-in mice on the two different genetic backgrounds were mated for homozygosity. *Pkd2*^+/+^, *Pkd2*^+/poreL1^ and *Pkd2*^poreL1/poreL1^ were born at the expected Mendelian frequency and showed equal survival rates until over 12 months of age, thus demonstrating that the polycystin-2^poreL1^ protein was able to substitute for wild-type polycystin-2.

**Fig. 5.**
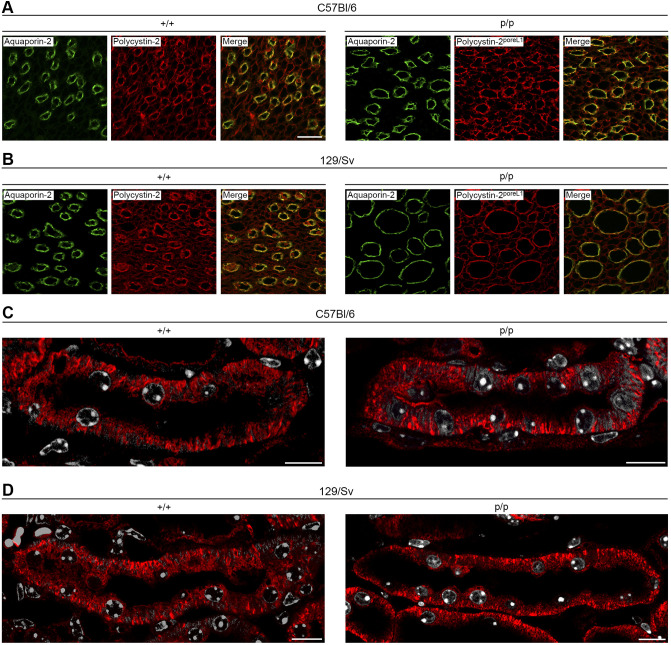
**Intrarenal distribution of polycystin-2^poreL1^ in homozygous *Pkd2*^poreL1^ knock-in mice.** (A,B) Immunofluorescence staining for aquaporin-2 as a marker for collecting ducts on the one hand and for polycystin-2 and polycystin-2^poreL1^ on the other hand in 6-month-old wild-type (+/+) and homozygous *Pkd2*^poreL1^ knock-in (p/p) female mice demonstrates the presence of wild-type polycystin-2 and of polycystin-2^poreL1^ in papillary collecting ducts in both the C57Bl/6 and 129/Sv genetic backgrounds. Note the larger diameter of collecting ducts in the *Pkd2*^poreL1/poreL1^ mice. Scale bar: 50 µm. (C,D) Immunofluorescence staining of kidney sections from 6-month-old wild-type (+/+) and homozygous *Pkd2*^poreL1^ knock-in (p/p) mice. Both in the C57Bl/6 and in the 129/Sv background, identical distributions of the wild-type and mutant polycystin-2 proteins (red signal) are seen. Nuclei are shown in white. Images are representative of three experiments. Scale bars: 10 µm.

**Fig. 6.**
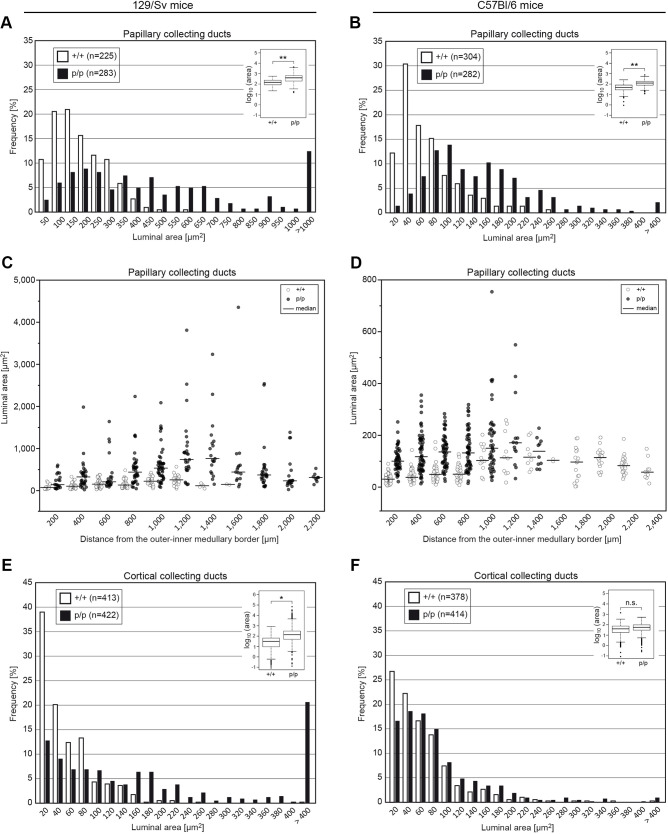
**Luminal areas of papillary and cortical collecting ducts in *Pkd2*^poreL1^ knock-in mice.** (A–D) In *Pkd2*^poreL1/poreL1^ knock-in (p/p) mice, the luminal area of papillary collecting ducts was larger than in wild-type (+/+) mice both on a 129/Sv and a C57Bl/6 background (A,B). This difference was observed independently of the position along the papillary axis (C,D; each circle represents an individual profile). The exclusive presence of collecting duct profiles closer to the papillary tip from knock-in mice on a 129/Sv background (and vice versa from wild-type mice on a C57Bl/6 background) is due to the analysis of one longer papilla. (E,F) In the case of cortical collecting ducts, the luminal area was only larger for *Pkd2*^poreL1/poreL1^ knock-in (p/p) mice on a 129/Sv but not on a C57Bl/6 background. Four wild-type and six homozygous *Pkd2^poreL1^* knock-in mice at 6 months of age were used for analysis. Box plots in insets summarize data of all profiles; boxes range from the 25th to the 75th percentile, the horizontal line in the box indicates the median, and whiskers extend to data within 1.5 times the interquartile range. *P<0.05; **P<0.01; n.s., not significant (linear mixed model).

We stained kidney sections of wild-type and homozygous knock-in mice and detected a prominent signal for polycystin-2 and polycystin-2^poreL1^ in collecting ducts (Fig. 5A,B), consistent with previously published results (Wu et al., 1998). Furthermore the intracellular distribution of the wild-type and mutant polycystin-2 proteins was indistinguishable (Fig. 5C,D). Since the *Pkd2* and *Pkd2*^poreL1^ genes are prominently expressed in collecting ducts we determined their luminal width in order to test our hypothesis that a polycystin-2 protein with altered ion channel properties leads to a larger tubular diameter (and eventually to renal cysts). It has been shown repeatedly that cortical and papillary collecting ducts differ both functionally and in their gene expression profile (Hinze et al., 2021; Ransick et al., 2019); therefore we discriminated between those segments to avoid any systematic errors. The immunofluorescence staining (Fig. 5A,B) had already suggested that the diameter of papillary collecting ducts was larger in homozygous *Pkd2*^poreL1^ knock-in mice than in wild-type mice, and careful morphometric measurements corroborated this observation for both genetic backgrounds (Fig. 6A, *P*=0.005, *t*=−3.69, d.f.=9; Fig. 6B, *P*=0.003, *t*=−4.07, d.f.=9). We want to emphasize that the mice were perfusion-fixed and that the same perfusion pressure was employed for all mice, therefore the observed differences cannot arise from randomly collapsed tubular structures. It should be noted that the luminal area of papillary collecting ducts was larger in wild-type 129/Sv than in wild-type C57Bl/6 mice to start with. In order to rule out that we were taken in by a positional bias of the collecting ducts along the papillary axis, we also sorted the profiles according to their distance from the outer-inner medullary border. This analysis demonstrated that the differences observed between the knock-in and wild-type mice were not due to an uneven sampling of the profiles in the papillae but were valid independently of the location (Fig. 6C,D). When the luminal area of cortical collecting ducts was investigated a difference between knock-in and wild-type mice was only detected on the 129/Sv background (Fig. 6E, *P*=0.029, *t*=−3.33, d.f.=4; Fig. 6F, *P*=0.22, *t*=−1.46, d.f.=4). In the case of distal nephron segments (thick ascending limb, distal convoluted tubule) no statistically significant difference was seen between knock-in and wild-type mice on the 129/Sv background (Fig. S6A, *P*=0.08, *t*=−2.25, d.f.=4; Fig. S6B, *P*=0.45, *t*=−0.78, d.f.=10), which is why we refrained from further characterizing the knock-in mice on the C57Bl/6 background.

### *Pkd2*^poreL1^ knock-in mice develop kidney cysts in a strain-dependent fashion

**Fig. 7.**
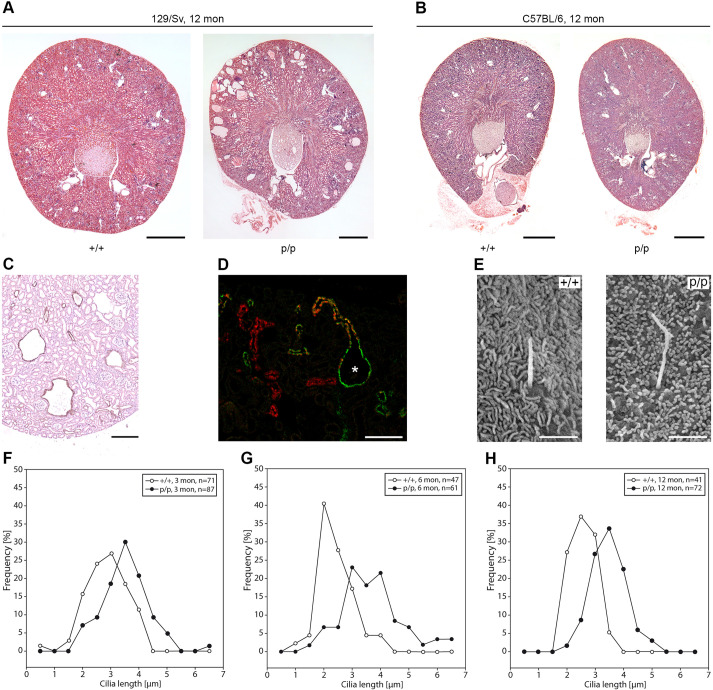
**Polycystic kidney disease in *Pkd2*^poreL1^ knock-in mice.** (A,B) Transverse kidney sections from 12-month-old wild-type (+/+) and homozygous *Pkd2*^poreL1^ knock-in (p/p) were stained with H&E. Whereas kidney cysts can be easily seen at the age of 12 months on a 129/Sv background, no kidney cysts were observed on a C57Bl/6 background over a period of 12 months. Scale bars: 1 mm. (C,D) Immunohistochemical staining of kidney sections from homozygous *Pkd2*^poreL1^ knock-in mice on a 129/Sv background for aquaporin-2 (a marker of principal cells in collecting ducts) shows that cysts originate in collecting ducts (C). Double-immunofluorescence staining for aquaporin-2 (green) and calbindin (a marker of connecting tubules, red) demonstrates the sudden transition from a calbindin-positive connecting tubule to an aquaporin-2-positive collecting duct cyst (asterisk in D). Scale bars: 100 µm. (E–H) Scanning electron micrographs were taken from kidneys of 3-, 6- and 12-month-old wild-type and *Pkd2*^poreL1^ knock-in mice of both sexes on a 129/Sv background to determine the length of primary cilia. No obvious morphological alterations of primary cilia in collecting ducts were detected (E). Scale bars: 2 µm. At all three ages the length of primary cilia in the knock-in mice is shifted towards higher values. The number of cilia analyzed is given in the upper right-hand corner of the line graphs in panels F–H. Images are representative of at least three animals per condition.

In addition to enlarged collecting ducts, kidney sections of *Pkd2*^poreL1/poreL1^ mice on a 129/Sv background also already contained sporadic tubular cysts at 1 month of age and an increased number of cysts at 12 months (Fig. 7A). However, we never detected kidney cysts in *Pkd2*^poreL1/poreL1^ mice on a C57Bl/6 background (Fig. 7B). Staining with antibodies against aquaporin-2 demonstrated that cysts developed in collecting ducts (Fig. 7C,D). Since it is generally acknowledged that primary cilia are involved in cystogenesis, we also measured the length of primary cilia in collecting ducts of 3-, 6- and 12-month-old mice by scanning electron microscopy. Primary cilia in wild-type mice were between 0.5 and 4.0 µm long, and the distribution of cilia length peaked between 2.0 and 3.0 µm. In homozygous knock-in mice, however, the length of primary cilia was increased at all ages (Fig. 7E–H). It is still poorly understood how cells measure and regulate ciliary length. The transcription factor Foxj1 (formerly known as Hfh-4) has been shown to positively regulate the length of primary cilia (Cruz et al., 2010; Lu et al., 2015), and indeed we found higher mRNA levels for Foxj1 in collecting ducts isolated from homozygous *Pkd2*^poreL1^ knock-in mice than in wild-type mice (Fig. S7A). In contrast, mRNA levels for the microtubule-associated protein Nde1 were lower in the knock-in mice (Fig. S7B) which is consistent with the fact that Nde1 has an inhibitory effect on the length of primary cilia (Inaba et al., 2016; Kim et al., 2011). Nde1 is believed to mediate its effect at least partially through its interaction with the dynein light chain Dynll1 (also known as LC8) (Kim et al., 2011) but the mRNA levels for Dynll1 were not different between wild-type and knock-in mice (Fig. S7C). These results suggest that the upregulation of Foxj1 and the downregulation of Nde1 contributes to the increased length of primary cilia in the *Pkd2*^poreL1^ knock-in mice.

**Fig. 8.**
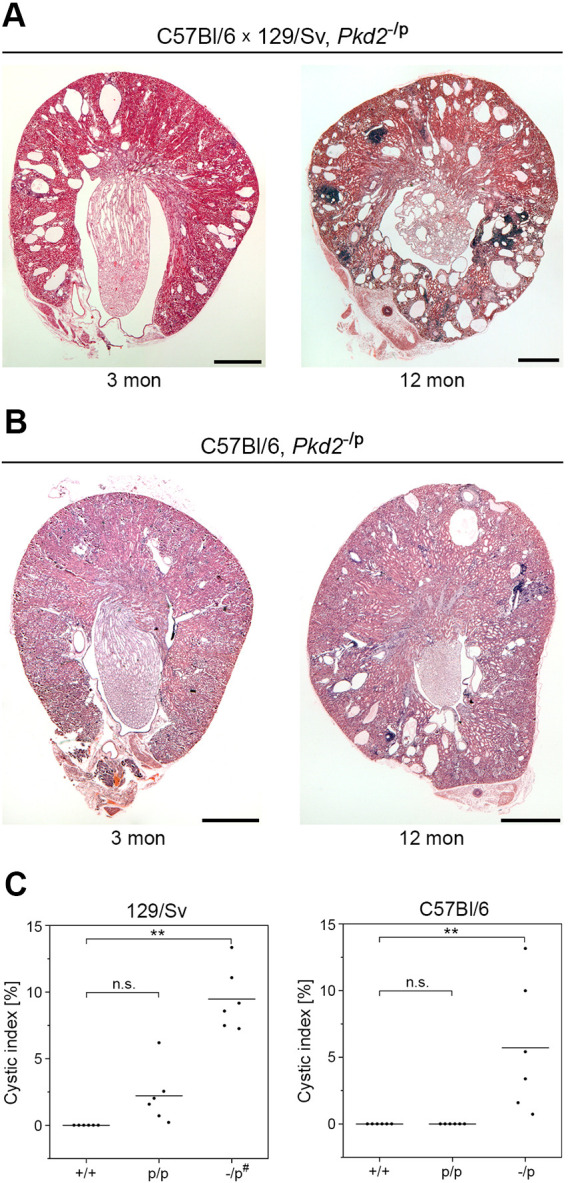
**Deletion of the wild-type *Pkd2* allele in combination with the *Pkd2*^poreL1^ allele enhances cystogenesis.** (A,B) Compound heterozygous *Pkd2* mice containing a *Pkd2* null allele (−) and the *Pkd2*^poreL1^ knock-in allele (p) show accelerated cyst formation. Images show transverse kidney sections stained with H&E. On a mixed 129/Sv and C57Bl/6 background, cyst formation was already seen at 3 months (A), and, on a C57Bl/6 background, kidney cysts were observed at 12 months of age (B). Scale bars: 1 mm. (C) Cystic indices of 12-month old mice with the indicated genetic backgrounds, both sexes were used for the analysis. #, mixed 129/Sv and C57Bl/6 background. **P<0.01 (one-way ANOVA using Tukey's post-hoc test); the horizontal lines represent the mean values.

Cyst formation in the homozygous knock-in mice on a 129/Sv background was very consistent but it proceeded slowly, and we therefore decided to mate the *Pkd2*^+/poreL1^ mice with heterozygous *Pkd2*-null mice (Wu et al., 1998, 2000). In compound heterozygous *Pkd2*^−/poreL1^ mice no wild-type *Pkd2* allele and only one *Pkd2*^poreL1^ allele will be present, so that these animals may be more prone to developing kidney cysts than the *Pkd2*^poreL1/poreL1^ mice. Indeed cystogenesis in the compound *Pkd2*^−/poreL1^ mice proceeded more rapidly than in the homozygous *Pkd2*^poreL1/poreL1^ mice on a mixed (50%/50%) C57Bl/6×129/Sv background (Fig. 8A), and cysts were now identifiable on kidney sections of compound heterozygous *Pkd2*^−/poreL1^ mice even on the C57Bl/6 background (Fig. 8B). The differences between the various genotypes also became clear when we determined the cystic index. For both genetic backgrounds, the difference between *Pkd2*^−/poreL1^ mice and *Pkd2*^+/+^ mice was statistically highly significant whereas this was not the case for the difference between *Pkd2*^poreL1/poreL1^ mice and *Pkd2*^+/+^ mice (Fig. 8C). Although somewhat surprising at first sight for the *Pkd2*^poreL1/poreL1^ mice on a 129/Sv background, the lack of statistical significance becomes clear from the fact that only a small number of cysts develop in those animals.

In both the *Pkd2*^poreL1/poreL1^ and the *Pkd2*^−/poreL1^ mice, the mutations in the *Pkd2* gene are present in the germline and will therefore have an impact on the process of tubulogenesis from the earliest stages onwards (i.e. in establishing the tubular diameter). In order to examine whether the pore mutant also had an effect at a later stage of tubular differentiation we generated *Pkhd1*::Cre; *Pkd2*^lox/poreL1^ mice by mating *Pkhd1*::Cre; *Pkd2*^+/lox^ with our *Pkd2*^+/poreL1^ mice. The promoter of the *Pkhd1* drives gene expression in differentiated collecting ducts (Williams et al., 2014) and therefore allows inactivation of a floxed *Pkd2* allele from the Cre recombinase specifically therein. Whereas *Pkhd1*::Cre; *Pkd2*^lox/lox^ mice had developed massive cysts at 4 weeks of age, we could not detect any kidney cysts in *Pkhd1*::Cre; *Pkd2*^lox/poreL1^ mice at the same age (data not shown). This argues that one *Pkd2*^poreL1^ allele is able to substitute for wild-type *Pkd2* at a later developmental stage. Alternatively, it is also possible that it takes (much) longer for cysts to develop in *Pkhd1*::Cre; *Pkd2*^lox/poreL1^ mice.

## DISCUSSION

### Distinct electrophysiological properties of polycystin-2, polycystin-2^poreL1^ and polycystin-2L1 can be explained by structural differences

The electrophysiological properties of polycystin-2, polycystin-2L1 and the chimeric channel polycystin-2^poreL1^ were investigated in the *Xenopus laevis* oocyte expression system. Our data indicate that polycystin-2 is significantly more permeable to K^+^ than to Na^+^ ions, which is consistent with selectivity data reported for a gain-of-function polycystin-2 mutant channel in which the phenylalanine residue at position 604 is mutated to proline (Arif Pavel et al., 2016) and for polycystin-2 currents recorded from primary cilia (Liu et al., 2018). Although the ionic radii suggest that both ions should permeate through the selectivity filter equally, the hydration radii show considerable size differences (Tables S2 and S3). It is remarkable that the radius at the position of amino acid L641 in the selectivity filter of polycystin-2 coincides with the radius of hydrated K^+^, thus indicating an ideal coordination of this ion. The radius of hydrated Na^+^ is markedly larger and the hydration shell holds tighter to the ion compared with K^+^, as indicated by the hydration free energy. This observation strongly supports the preference of K^+^ over Na^+^ in polycystin-2. In contrast, we found that polycystin-2L1 is equally permeable for Na^+^ and K^+^ ions, which is in good agreement with previously reported data (Chen et al., 1999). The chimeric channel polycystin-2^poreL1^ shows an intermediate phenotype. Its permeability for K^+^ ions is slightly higher than that for Na^+^ ions but, compared to polycystin-2, this difference is much less pronounced. Moreover, polycystin-2^poreL1^ is slightly less permeable for Li^+^ ions than for Na^+^ ions, unlike polycystin-2 and similar to polycystin-2L1. In our model, the amino acids in the selectivity filter of polycystin-2^poreL1^ do not lead to constrictions that are compatible with the radii of hydrated K^+^, Na^+^ and Li^+^ ions. Therefore neither K^+^ nor Na^+^ or Li^+^ ions can be specifically coordinated. The permeability of those ions rather seems to correspond to their hydration free energy; for example, the low permeability for Li^+^ ions correlates with the high energy required to remove their hydration shell, and vice versa for K^+^ ions (Table S3). Thus, replacing the pore region of polycystin-2 by that of polycystin-2L1 makes the permeability profile of polycystin-2^poreL1^ for K^+^, Na^+^ and Li^+^ ions similar to that of polycystin-2L1.

It is controversial whether polycystin-2 is a Ca^2+^-permeable non-selective cation channel, but that possibility has been discussed (Douguet et al., 2019). Our experiments with Ca^2+^ as the sole cation in the bath solution did not provide evidence for polycystin-2-mediated Ca^2+^ influx, which is consistent with recent findings obtained in primary cilia showing that polycystin-2 is poorly permeable for Ca^2+^ ions (Liu et al., 2018). Moreover, recent data obtained in the oocyte expression system demonstrated that the current response elicited by a high Ca^2+^ concentration (70 mM) was observed only in oocytes expressing a gain-of-function polycystin-2 mutant channel (L677A/N681A) but not in oocytes expressing wild-type polycystin-2 or another gain-of-function mutant, polycystin-2 (F604P) (Wang et al., 2019). This further supports our conclusion that polycystin-2 expressed in oocytes has little if any Ca^2+^ permeability. The radius of the ion translocation pore in the selectivity filter corroborates this conclusion (Tables S2 and S3). However, our results do not rule out that polycystin-2 may conduct Ca^2+^ under certain physiological conditions that remain to be elucidated.

In contrast, there is evidence that polycystin-2L1 is highly permeable for Ca^2+^ ions (DeCaen et al., 2013, 2016) which is in agreement with the architecture of its pore loop. The large inward current response we observed in oocytes expressing polycystin-2L1 when they were exposed to a high extracellular Ca^2+^ concentration supports the concept that polycytin-2L1 is permeable to Ca^2+^. Importantly, we saw a similar response in oocytes expressing polycystin-2^poreL1^, which shows that replacing the pore region of polycystin-2 by that of polycystin-2L1 increases the permeability of polycystin-2^poreL1^ to Ca^2+^ ions. This conclusion is further supported by findings from a previous study that used the opposite approach (Shen et al., 2016). In that study, it was shown that replacing the pore region of polycystin-2L1 by that of polycystin-2 significantly reduced the permeability of the polycystin-2L1 mutant channel to Ca^2+^ ions. It is conceivable that the increased distance between the L641 and D643 residues in the selectivity filter of our polycystin-2^poreL1^ mutant not only explains the reduced inhibitory effect of Ca^2+^ on the channel but also facilitates Ca^2+^ permeation through the pore. Moreover, it has been shown that the loss of a negative charge (replacement of an aspartate residue at position 525 by asparagine) in the vicinity of the selectivity filter of polycystin-2L1 dramatically reduces its Ca^2+^ permeability (DeCaen et al., 2016). Similar to the situation in the polycystin-2L1 (D525N) mutant channel, the analogous position in polycystin-2 is occupied by a neutral asparagine residue (N645) close to the selectivity filter. It is also noteworthy that by replacing the pore region of polycystin-2 with that of polycystin-2L1, an asparagine residue instead of aspartate is introduced at position 625 and an aspartate residue instead of asparagine is introduced at position 645. The inverse arrangement of these two amino acids probably leads to their loss of interaction due to the fact that N625 in polycystin-2^poreL1^ is fully engaged with T635 and therefore is positioned too far away to interact with D645 (Fig. 3F). Thus, the more restricted pore opening in polycystin-2 is replaced by a wider pore in polycystin-2^poreL1^, which may provide an additional explanation for the increased Ca^2+^ permeability of polycystin-2^poreL1^ compared to polycystin-2.

We also found that polycystin-2 is strongly inhibited by divalent cations, which is consistent with a similar effect seen with the F604P mutant of polycystin-2 (Arif Pavel et al., 2016). The cryo-electron microscopical structure of polycystin-2 suggests that the movement of ions through the channel is blocked by Ca^2+^ ions due to the binding of Ca^2+^ to the negatively charged D643 residues within the selectivity filter (Wilkes et al., 2017). The increased distances between opposing D523 residues of the respective protomers of polycystin-2L1 (Su et al., 2018b), and presumably also in polycystin-2^poreL1^, may alleviate the inhibition of these channels by Ca^2+^ ions.

Although it has been demonstrated that polycystin-1 and polycystin-2 form a heterotetrameric complex with a 1:3 stoichiometry (Su et al., 2018a) it is still unclear how polycystin-1 affects the channel function of polycystin-2. An earlier publication reported that, in the oocyte expression system, polycystin-1 inhibited the activity of the polycystin-2 (F604P) mutant and altered the ion channel properties of another gain-of-function mutant protein, polycystin-2 (L677A/N681A) (Wang et al., 2019). In contrast, a subsequent study in HEK 293 cells described a stimulatory effect of polycystin-1 on polycystin-2 (F604P), but not on wild-type polycystin-2 (Ha et al., 2020). Moreover, it is still a matter of debate whether heteromeric complexes between polycystin-1 and polycystin-2 play an important physiological role because it has been shown that polycystin-2-dependent ion channel activity in primary cilia does not require the presence of polycystin-1 (Liu et al., 2018). In light of these conflicting findings, we did not attempt to compare a possible functional interaction of polycystin-1 with polycystin-2 and polycystin-2^poreL1^, respectively, in the oocyte system.

Taken together our electrophysiological data demonstrate that replacing the pore region of polycystin-2 with that of polycystin-2L1 alters the selectivity of the resulting polycystin-2^poreL1^ channel for K^+^, Na^+^ and Li^+^ ions and increases its permeability for Ca^2+^ ions. This latter effect may be particularly important because a dysregulation of intracellular Ca^2+^ homeostasis is widely hypothesized to be a major factor in the pathogenesis of ADPKD (Brill and Ehrlich, 2020). However, the mechanistic link on how the altered ion channel properties of polycystin-2^poreL1^ cause tubular dilation and cyst formation in the knock-in mouse model is presently elusive. The main reason for this is that the (patho-)physiological role of polycystin-2 is still poorly understood and complicated by the fact that polycystin-2 may have different functions depending on its subcellular localization.

### The phenotype of the Pkd2^poreL1^ knock-in mice mimics mild forms of ADPKD

Our study strongly supports an additional pathogenetic mechanism of cyst formation. Not only may a loss of polycystin-2 be responsible for cystogenesis (Wu et al., 1998) but cystic kidney disease may also result from the altered ion channel properties of a mutated polycystin-2 protein. Since we were able to demonstrate that polycystin-2^poreL1^ enters the primary cilium, a trafficking defect, as has been shown for other polycystin-2 mutant proteins (Cai et al., 2014; Walker et al., 2019; Yoshiba et al., 2012), is unlikely to explain cyst formation in our case.

In our homozygous *Pkd2*^poreL1^ knock-in mice, the width of collecting ducts extends over a wider range (up to the formation of full-blown cysts) than the width of collecting ducts in adult wild-type mice. The development of collecting ducts with wider lumina is accompanied by the formation of longer primary cilia. While longer cilia were observed from 3 to 12 months after birth, with a very similar size distribution at each time point examined, the severity of cystic kidney disease increased with age. Therefore it appears that the development of longer primary cilia precedes cyst formation, but admittedly we cannot provide firm experimental evidence for such a conclusion. It is curious that kidney cysts in *Pkd2*^poreL1/poreL1^ mice developed on a 129/Sv and not on a C57Bl/6 background, indicating that modifying effects are of great importance when polycystic kidney disease results from a mutation in the *Pkd2* gene. Since the width of collecting ducts in wild-type 129/Sv mice is larger than in wild-type C57Bl/6 mice to start with, it is possible that the control circuit determining collecting duct diameter in 129/Sv mice is already more susceptible to cyst-promoting changes. Cyst formation was markedly accelerated when we combined a *Pkd2*-null allele with the *Pkd2*^poreL1^ allele. In those compound heterozygous mice, we were also able to detect renal cysts in C57Bl/6 mice, albeit only at 12 months of age. Obviously the polycystin-2^poreL1^ mutant protein can substitute for the function of wild-type polycystin-2 to a large extent but not completely, otherwise no cysts would develop.

In the *Pkd2*^poreL1/poreL1^ mice, the mutated *Pkd2* gene is present in all cells of the kidneys. Despite this fact cyst formation was observed only focally, and we detected no cysts in the liver and pancreas. It is possible that other – so far unknown – confounding factors determine whether renal tubules expand into cysts; a two-hit mechanism (Pei, 2001) cannot explain the focal nature of cysts when both alleles of the *Pkd2* gene are mutated in the germline. In the kidneys, the *Pkd2* gene is most strongly expressed in the collecting ducts (Wu et al., 1998), which correlates with the site of cyst formation. This leads to the question of whether polycystin-2 is not essential in other parts of the kidney, such as the proximal tubules, or if it is present, why the *Pkd2* gene is expressed there at much lower levels. There is evidence that collecting ducts are more prone to cyst formation than other tubular structures in the kidney (Fedeles et al., 2011). We cannot rule out, however, that the importance of polycystin-2 is not the same in all tubular compartments of the kidneys despite the fact that lumen formation has to be controlled at all those sites.

More than 200 likely and highly likely pathogenic mutations have been described in the *PKD2* gene, including 28 missense mutations (https://pkd.mayo.edu/). Conflicting evidence has been reported regarding the effect of these missense mutations, and their functional characterization remains incomplete (Arif Pavel et al., 2016; Vien et al., 2020; Zheng et al., 2018). Interestingly, three ADPKD-associated mutations are localized within the pore domain and affect highly conserved residues of polycystin-2 (F629S, C632R and R638C). Two of them (F629S and R638C) have been shown to abolish the activity of the polycystin-2 (F604P) mutant protein (Arif Pavel et al., 2016), but we are not aware of any studies demonstrating the effect of these mutations on wild-type polycystin-2. In addition, missense mutations within the polycystin/TOP domain of polycystin-2 also cause a loss of channel function in primary cilia of transfected HEK 293 cells (Vien et al., 2020). In light of these disease-causing single amino acid changes, it is somewhat surprising that we did not observe a more-severe phenotype when we exchanged almost the entire pore domain of polycystin-2 with that of polycystin-2L1. Our homozygous *Pkd2^poreL1^* knock-in mice are viable and only show a renal phenotype, in contrast to what is seen for the homozygous *Pkd2* knock-out mice, which die *in utero* and also develop cysts in the pancreas and liver (Wu et al., 1998, 2000). Furthermore the homozygous *Pkd2*^poreL1^ knock-in mice only develop renal cysts on a 129/Sv and not a C57Bl/6 background, and even the combination of the knock-in mutation with a null mutation did not lead to an increased lethality up to 12 months of age. Accordingly our *Pkd2*^poreL1^ allele can be classified as a hypomorphic mutant. It may be that the insertion of a functional pore domain, albeit with different electrophysiological properties (in the case of the polycystin-2^poreL1^ mutant protein), serves the role of polycystin-2 better than the overall disruption of its function, as in the case of the missense mutations affecting single amino acids. It is tempting to speculate that at least some of the *PKD2* mutations observed in ADPKD patients do not completely abolish the function of polycystin-2 but rather alter its ion channel properties, thereby inducing cyst formation to an extent similar to that observed in our mouse model.

## MATERIALS AND METHODS

### Expression plasmids

The full-length human PKD2 cDNA was cloned into the mammalian expression plasmid pUHD 10-3 (kind gift from Hermann Bujard, Molekulare Biologie der Universität Heidelberg, Heidelberg, Germany) together with a DNA fragment encoding a HA-epitope tag at the COOH-terminus of polycystin-2. The pore mutant was generated by site-directed mutagenesis with the oligonucleotides 5′-GGCACTCAGGTCGAGAATTTCAGTACTTTCATAAAGTGTATCTTCACTC-3′, 5′-ATCATTTTGGGCGATTTCGACTATAACGCGATTGACAACGCTAATCGAGTTTTG-3′, and 5′-CAAAACTCGATTAGCGTTGTCAATCGCGTTATAGTCGAAATCGCCCAAAATGAT-3′. For functional expression in *Xenopus laevis* oocytes, constructs coding for full-length human polycystin-2 (untagged) and polycystin-2L1 (with a C-terminal HA-epitope tag) were subcloned into the plasmid pTLN (Lorenz et al., 1996). Deletion of the 34-amino-acid domain extending from E787 to S820 and the pore mutation were introduced using the QuikChange Lightning site-directed mutagenesis kit (Agilent, TX, USA) with the following primers: poreL1, forward: 5′-TGGCACTCAGGTCGAGAATTTCAGTACTTTCATAAAGTGTATCTTCACTCAATTCCGTATCATTTTGGGCGATTTCGACTATAACGCGATTGACAACGCTAATCGAGTTTT G-3′; poreL1, reverse: 5′-TTCCTCAATCTCTGCAAAGTTGATATCGCCCAAAATGATACGGAATTGAGTGAAGATACACTCTTGGAAAGTACTGAAGTCATC-3′; 34-amino-acid deletion, forward: 5′-CGACTTGGAGAAAGAGAGGGGACATAGCTCCAGAAGG-3′, 34-amino-acid deletion, reverse: 5′-CCTTCTGGAGCTATGTCCCCTCTCTTTCTCCAAGTCG-3′. All constructs were confirmed by sequence analysis. Linearized plasmids were used as templates for cRNA synthesis using T7 RNA polymerase (mMessage mMachine, Ambion, Austin, TX, USA).

### Two-electrode voltage-clamp and biotinylation experiments in the *Xenopus laevis* oocyte expression system

Isolation of oocytes and two-electrode voltage-clamp experiments were performed essentially as described previously (Haerteis et al., 2009; Ilyaskin et al., 2018; Rauh et al., 2010). Female *Xenopus laevis* were anesthetized with 0.2% ethyl 3-aminobenzoate methanesulfonate (MS-222) (Merck, cat. no. E10521), and ovarian lobes were obtained by a small abdominal incision. Defolliculated stage V–VI oocytes were injected with 20 ng of cRNA encoding polycystin-2, polycystin-2^poreL1^ and polycystin-2L1. To suppress the expression and possible interference of endogenous connexin38 hemichannels an established approach (Bahima et al., 2006; Ebihara, 1996; Ilyaskin et al., 2021) was used, that is 3 ng of an antisense phosphothioate oligomer corresponding to nucleotides −5 to +25 relative to the coding region of connexin38 (5′-GCTTTAGTAATTCCCATCCTGCCATGTTTC-3′) (biomers.net, Germany) were co-injected together with the cRNAs for polycystin-2, polycystin-2^poreL1^ and polycystin-2L1. Oocytes injected only with the antisense DNA against connexin38 were used as control oocytes. Injected oocytes were incubated in ND96 solution (96 mM NaCl, 2 mM KCl, 1.8 mM CaCl_2_, 1 mM MgCl_2_, 5 mM HEPES, adjusted to pH 7.4 with Tris) supplemented with 100 units/ml of sodium penicillin and 100 µg/ml of streptomycin sulphate to inhibit bacterial growth. Measurements were performed at 48 to 96 h after cRNA injection. A modified ND96 solution was used as the standard NaCl bath solution (96 mM NaCl, 4 mM KCl, 1 mM CaCl_2_, 1 mM MgCl_2_, 10 mM HEPES, adjusted to pH 7.4 with Tris). To obtain a NaCl bath solution nominally free of divalent cations, CaCl_2_ and MgCl_2_ were excluded from the ND96 solution. Additional modified ND96 solutions without divalent cations were obtained by replacing 95 mM NaCl with the same concentration of NMDG-Cl, LiCl or KCl. In order to investigate the Ca^2+^ permeability of the channels, a bath solution containing only Ca^2+^ as a permeable cation was used (50 mM CaCl_2_, 10 mM HEPES, adjusted to pH 7.4 with Tris). A similar MgCl_2_ bath solution was used as control (50 mM MgCl_2_, 10 mM HEPES, adjusted to pH 7.4 with Tris). Bath solution exchanges with a gravity-driven system were controlled by the ALA BPS-8 magnetic valve system in combination with a TIB14 interface (HEKA, Lambrecht, Germany). An individual oocyte was placed in an experimental chamber close to the site of inflow where it was fixed by the impaling microelectrodes. Experiments were performed at room temperature. Oocytes were held continuously at a holding potential of −60 mV unless indicated otherwise using an OC-725C amplifier (Warner Instruments Corp., Hamden, USA). Current–voltage (*I*/*V*) plots were obtained from voltage-step protocols using consecutive 1000 ms step changes of the clamp potential from −60 to −120 mV up to +60 mV in increments of 20 mV (PULSE and LIH1600, HEKA). The average current values reached during the last 300 ms of the voltage steps were used for the *I*/*V* plots.

The permeability ratios for monovalent cations, *P*_X_^+^/*P*_Na_^+^, were calculated using the following equation (Hille, 2001):(1)
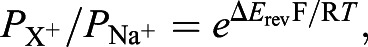
where Δ*E*_*rev*_ is the shift of the reversal potential (*E*_rev_) caused by replacing Na^+^ in the bath solution with cation X^+^ (Δ*E*_rev_=*E*_rev,X_−*E*_rev,Na_), F is Faraday's constant, R is the universal gas constant, and *T* is the absolute temperature.

Cell surface proteins were labeled with biotin essentially as described in a previous publication (Krueger et al., 2018). Briefly, oocytes were treated with EZ-Link Sulfo-NHS-SS-Biotin (Pierce) and lysed by mechanical shearing through a needle before biotinylated proteins were precipitated with NeutrAvidin beads (Pierce). Precipitated proteins were subjected to western blot analysis with the polyclonal rabbit anti-polycystin-2 antiserum YCB9 (diluted 1:10,000) (Wu et al., 1998) and a polyclonal rabbit anti-β-actin antiserum (Merck, cat. no. A2066; diluted 1:5000) as primary antibodies, followed by a horseradish peroxidase-conjugated goat anti-rabbit-IgG antibody (Santa Cruz Biotechnology, cat. no. sc-2054; diluted 1:50,000) as secondary antibody.

### Comparative modeling of polycystin-2^poreL1^

The structural model of polycystin-2^poreL1^ was obtained using the program Modeller v9.17 (Šali and Blundell, 1993) and was based on the monomeric structure coordinates of wild-type human polycystin-2 (Wilkes et al., 2017) (PDB ID: 5MKF), murine polycystin-2L1 (Su et al., 2018b) (PDB ID: 5Z1W) and human polycystin-2L1 (Hulse et al., 2018) (PDB ID: 6DU8) as templates. An initial model was generated by default modeling in the ‘automodel-class’ suite of Modeller, such that the sequence of polycystin-2^poreL1^ was threaded into the structural coordinates of wild-type polycystin-2 and optimized for stereochemical requirements. This initial model was then used for multi-reference modeling of polycystin-2^poreL1^ based on the structure coordinates of wild-type polycystin-2 together with the structure coordinates of murine and human polycystin-2L1. Multi-reference modeling has the advantage of a broader conformational space for the target structure. The modeling procedure was set to utilize the modeling schedule with ‘very thorough variable target function’ (VTMF) and slow MD annealing (Šali and Blundell, 1993), thus generating model sets of 20 to 100 structures. Model quality was assessed by the discrete optimized protein energy (DOPE) potential (Shen and Sali, 2006). The first 15 models of each set were analyzed visually, improved manually in the Coot environment (Emsley et al., 2010), and the best resulting model was used as the initial model for the next round of modeling until the models converged.

### Genetically engineered mouse models

The targeting construct was generated by standard molecular cloning techniques using restriction enzymes (Ausubel et al., 1996) and by PCR using a BAC clone (RP22-21I14) harboring the murine *Pkd2* gene as the template. This BAC was derived from the 129S6/SvEvTac mouse strain and obtained from the BACPAC Resource Center. A floxed neomycin resistance cassette was introduced into the *Xma*I site in the intron between exons 8 and 9. The altered pore region was generated by site-directed mutagenesis using the oligonucleotides 5′-CGGCACCCAGGTCGAAAACTTCAGCACTTTCGTGAAATGTATGTAAGTATC-3′, 5′-AACTATGCCAGCATAGGATCCATCTTTAGTAAGCG-3′, 5′-ATCATTTTGGGTGATTTCGACTACAACGCCATCGATAACGCTAACCGAGTT-3′, and 5′-CAAAACTCGGTTAGCGTTATCGATCGCGTTGTAGTCGAAATCACCCAAAAT-3′. When constructs were generated with the help of restriction enzymes, only the cloning sites were sequenced, in the case of PCR-generated constructs, the complete PCR product was sequenced. The final targeting construct was cloned into the AgeI and BstBI sites of the plasmid Litmus28i (New England Biolabs) and used to transfect the R1 embryonic stem cell line derived from the 129/Sv mouse strain. Once germline transmission was established, heterozygous offspring were backcrossed to C57Bl/6J and 129S2/SvPasCrl mice, respectively.

In order to differentiate between the wild-type and mutant *Pkd2* alleles, 10 µg of genomic DNA isolated from mouse tail biopsies was digested with *Bam*HI, separated by agarose gel electrophoresis, transferred onto a membrane by Southern blotting and hybridized with a DNA fragment generated from exon 6 (5′ probe) or exon 13 (3′ probe). Fragment sizes were 19 kbp for the wild-type allele (5′ and 3′ probe), 8.5 kbp (5′ probe) and 10.5 kbp (3′ probe) for the mutant alleles. After Cre-mediated removal of the neomycin resistance cassette oligonucleotides flanking the remaining loxP site (5′-CAAGCCGTGTTGAGATGTTGG-3′, 5′-TGTCTCCTAGAAGTGGAAACC-3′) were used for PCR-mediated genotyping, thus generating PCR products of ∼300 bp (wild-type allele) and ∼400 bp (mutant allele).

*Pkd2*^+/−^ (Wu et al., 1998, 2000), *Pkhd1*::Cre (Williams et al., 2014) and *Pkd2*^+/lox^ (Nishio et al., 2010) mice have been described previously.

### RNA isolation and quantitative PCR analysis

Total RNA was prepared using the Isolate II RNA mini kit (Bioline), then cDNA was generated with the iScript cDNA synthesis kit (Bio-Rad) according to the manufacturers’ protocols. Quantitative real-time PCR was performed with the SensiFAST™ SYBR^®^ No-ROX Kit (Bioline) in the Roche LightCycler^®^ 480 (Roche Diagnostics). After the cDNA was denatured for 7 min at 95°C, 35 PCR cycles (20 s at 94°C, 20 s at 64°C and 10 s at 72°C) followed. The following oligonucleotides were used: FoxJ1_forward, 5′-ACCCTACTCCTATGCCACTCTCAT-3′; FoxJ1_reverse, 5′-TGCATGGCGGAAGTAGCAGAAGTT-3′, Dynll1_forward, 5′-GGCCCATATCAAGAAGGAGTTTG-3′; Dynll1_reverse, 5′-GGATCACTGGGTGTTTGGCA-3′; Nde1_forward, 5′-AGATCTGCGGCAGGAATTGG-3′; Nde1_reverse, 5′-GGAGCTGTCCAGACCACG-3′; lamin A/C_forward, 5′-TGACTTGGTGTGGAAGGC G-3′; lamin A/C_reverse, 5′-CAGTGGAGTTGATGAGAGCGG-3′.

### Isolation of collecting ducts

Both kidneys were rapidly removed from anesthetized mice and transferred to ice-cold isolation buffer (140 mM NaCl, 0.4 mM KH_2_PO_4_, 1.6 mM K_2_HPO_4_, 1 mM MgSO_4_, 10 mM NaAcetate, 1.3 mM calcium gluconate, 1 mM α-ketoglutaric acid pH 7.4). Then the kidneys were perfused through the renal artery with 3 ml of a solution containing 5 mM glycine, 50 µg/ml of collagenase II (Merck, cat. no. C6885), 50 µg/ml of trypsin inhibitor type II-S (Merck, cat. no. T9128), and 25 µg/ml of DNase I (Merck, cat. no. DN25) in isolation buffer pre-warmed to 37°C (digestion buffer). When papillary collecting ducts were isolated, most of the cortex was removed until the papillae could be identified and isolated. When collecting ducts from whole kidneys, irrespective of their location, were isolated, the kidneys were cut into 4–5 transverse slices. Subsequently papillae and kidney slices were incubated in the digestion buffer but containing 500 µg/ml of collagenase II for 10 min at 37°C. The released tubules (fraction 1) were washed three times with ice-cold digestion buffer containing 7.5 mM bovine serum albumin (BSA) (sorting buffer). Thus, three additional fractions were collected and each digested for 5 min at 37°C in decreasing concentrations of collagenase II (dilution factor 1:2). Tubules were sorted in ice-cold sorting buffer at 60–100× magnification under a stereo microscope (ZEISS SteREO Discovery.V12) with continuous cooling at 4°C for a maximum of 2 h. Finally, collecting ducts were pelleted at 500 ***g*** and 4°C, then they were kept at −80°C until further preparation.

### Immunofluorescence staining of cells and tissue sections

Cells were fixed in 4% paraformaldehyde in 1× PBS for 20 min at room temperature. After three washes with 1× PBS, they were blocked and permeabilized for 45 min in 1× PBS, 2% BSA and 0.2% Triton X-100. After washing the cells once in 1× PBS, they were incubated for 1 h at room temperature with the following primary antibodies diluted in 1× PBS with 2% BSA: the rat monoclonal anti-HA-epitope antibody 3F10 (Roche, cat. no. 11 867 423 001; diluted 1:1000), a rabbit polyclonal anti-Arl13b antibody (Proteintech, cat. no. 17711-1-AP; diluted 1:2000), and the mouse monoclonal anti-γ-tubulin antibody GTU-88 (Merck, cat. no. T6557; diluted 1:2000). Subsequently the cells were washed once in 1× PBS with 350 mM NaCl, twice with 1× PBS, and incubated for 1 h at room temperature with the following secondary antibodies diluted in 1× PBS with 2% BSA: a Cy3-conjugated donkey anti-rat-IgG antibody (Jackson ImmunoResearch, cat. no. 712-165-150; diluted 1:600), an Alexa Fluor 488-conjugated goat anti-rabbit-IgG antibody (Thermo Fisher, cat. no. A11034; diluted 1:600), and a DyLight 405-conjugated donkey anti-mouse-IgG antibody (Rockland, cat. no. 610-746-124; diluted 1:100). After three washes in 1× PBS the cells were stained for 1 min with 10 μg/ml of Hoechst 33258 (Merck, cat. no. B2883). Finally, the cells were washed once with 1× PBS and mounted either in 1× PBS with 40% glycerol or in Mowiol. Images were taken with a LSM 710 confocal laser scanning microscope (Carl Zeiss, Inc.) and an Axiovert 200 fluorescence microscope (Carl Zeiss, Inc.).

Mice were perfusion-fixed with 1× PBS with 4% paraformaldehyde through the distal abdominal aorta for 3 min. Kidneys were removed, fixed with 1× PBS with 4% paraformaldehyde overnight, dehydrated and paraffin-embedded. Paraffin sections (5–7 µm thick) were deparaffinized, rehydrated and subjected to an antigen retrieval treatment if required (20 µg/ml of proteinase K in 50 mM Tris-HCl pH 8.0, 1 mM EDTA, 0.5% Triton X-100 for 10 min at room temperature or, alternatively, two 5 min microwave treatments in 10 mM sodium citrate, pH 6.0). After blocking in 1× PBS with 5% BSA for 30 min at room temperature, sections were incubated with the primary antibody diluted in 1× PBS with 1% BSA for 1 h at room temperature or overnight at 4°C. After washing in 1× PBS the sections were incubated with the secondary antibody diluted in 1× PBS with 1% BSA for 1 h at room temperature. Finally the sections were washed in 1× PBS, stained for 1 min with 10 μg/ml of Hoechst 33258, and mounted with Mowiol. The following primary antibodies were used: a mouse monoclonal anti-calbindin antibody (Merck, cat. no. C8666; diluted 1:4000), a polyclonal goat anti-aquaporin-2 antibody (Santa Cruz Biotechnology, cat. no. sc-9882; diluted 1:200), a rabbit polyclonal anti-laminin antibody (Merck, cat. no. L9393; diluted 1:100), a polyclonal rabbit anti-Na^+^/Cl^−^ cotransporter antiserum (diluted 1:1000) (Kim et al., 1998), a polyclonal rabbit anti-uromodulin antibody (Biotrend, cat. no. 8595-0004; diluted 1:100), and the polyclonal rabbit anti-polycystin-2 antibodies YCB9 and YCC2 (diluted 1:200) (Wu et al., 1998). The following secondary antibodies were used: an Alexa Fluor 488-conjugated donkey anti-goat-IgG antibody (Thermo Fisher, cat. no. A11055; diluted 1:600), an Alexa Fluor 568-conjugated donkey anti-goat-IgG antibody (Thermo Fisher, cat. no. A11057; diluted 1:600), an Alexa Fluor 568-conjugated donkey anti-mouse-IgG antibody (Thermo Fisher, cat. no. A10037; diluted 1:600), an Alexa Fluor 568-conjugated donkey anti-rabbit-IgG antibody (Thermo Fisher, cat. no. A10042; diluted 1:600), and a horseradish peroxidase-conjugated rabbit anti-goat-IgG antibody (Merck, cat. no. A5420; diluted 1:200).

### Histological analysis

Mice were perfusion-fixed with 1× PBS with 4% paraformaldehyde through the distal abdominal aorta for 3 min. Kidneys were removed, fixed with 1× PBS with 4% paraformaldehyde overnight, dehydrated and paraffin-embedded. Transverse paraffin sections (5–7 µm thick) sections were stained with hematoxylin and eosin (H&E) according to standard protocols.

To determine the luminal area of collecting ducts and distal tubules, kidneys were stained with antibodies against laminin, aquaporin-2, uromodulin and the Na^+^/Cl^−^ cotransporter as described above. The luminal areas of papillary and cortical collecting ducts, cortical thick ascending limbs and distal convoluted tubules were determined with the ImageJ software package. Only tubules with a roundness of at least 0.8, as determined by the laminin signal for the basement membrane, were analyzed.

### Scanning electron microscopy

Preparation of the kidneys for scanning electron microscopy was done according to Tanaka et al. (Tanaka et al., 1989) with modifications. Mice were perfusion-fixed with 1× PBS with 4% paraformaldehyde through the distal abdominal aorta for 3 min. Kidneys were cut in half and stored in 2% glutaraldehyde with 0.1 M sodium cacodylate pH 7.4 at 4°C until further processing. Samples were post-fixed in 1% OsO_4_ in the same buffer. After incubation in 30% dimethylformamide at 4°C overnight, freeze fracturing was performed with a cold knife under liquid nitrogen followed by immersion in dimethylformamide at room temperature for 1 h. After serial dehydration in increasing acetone concentrations, samples were dried at critical point (Leica EM CPD300), coated with platinum (Edwards Scancoat) and finally examined on a Zeiss LEO 1530 Gemini scanning electron microscope using the SE2 detector and the Smart SEM V05.06 software (Carl Zeiss Microscopy, Oberkochen, Germany). Measurements of ciliary length were performed with ImageJ.

### Statistical analysis

When the data followed a normal distribution, statistical significance was calculated according to Student's *t*-test, otherwise the Mann–Whitney *U* test was employed.

The luminal areas of the tubuli were compared between wild-type and knock-in mice by linear mixed models, which were calculated for each renal region (papillary collecting ducts, cortical collecting ducts, cortical thick ascending limbs and distal convoluted tubules) and genetic background (129/Sv and C57Bl/6) separately. To control for non-independence of measurements, we added a random intercept for section nested in individual, or only individual in cases where only one section per individual was evaluated. Luminal area was log_10_-transformed in all models to improve normality of the residuals. To control for heteroskedasticity in the residuals of the papillary collecting and cortical collecting ducts in 129/Sv mice, a constant variance function structure for treatment (wild-type versus knock-in mice) was added to allow for different variances in these two groups. The difference in the luminal area in collecting ducts of mice receiving water or maltose solution, respectively, was compared using a linear mixed model including a random intercept for individual. Areas were log_10_-transformed to improve normality in the residuals. All analyses were conducted in R version 4.0.2 (https://www.r-project.org/). Models were fit using the nlme package version 3.1 (https://CRAN.R-project.org/package=nlme).

### Ethics statement

All animal experiments were conducted in accordance with the German Animal Protection Law and were approved by the local government.

## Supplementary Material

Supplementary information
