# Supplementary material for: A polycystin-2 protein with modified channel properties leads to an increased diameter of renal tubules and to renal cysts
Source: J Cell Sci. 2021 Aug 23;134(16):jcs259013. doi: 10.1242/jcs.259013 (PMC8435292; doi:10.1242/jcs.259013)
Supplement: Supplementary information [file joces-134-259013-s1.pdf]

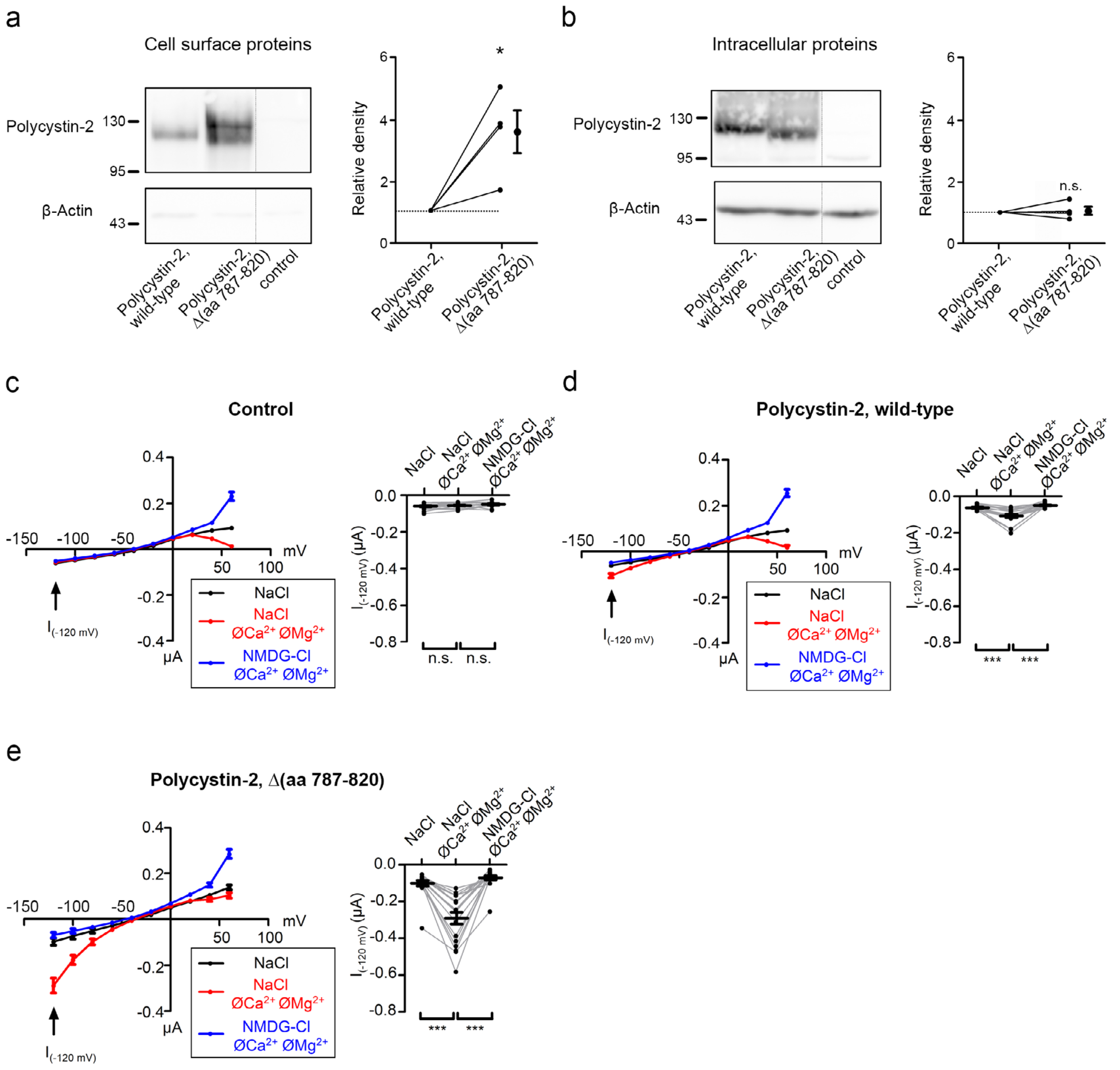

Fig. S1. Deletion of a 34-amino acid motif in the COOH-terminus of polycystin-2 increases its expression at the cell surface and enhances the polycystin-2-mediated inward current elicited by the removal of divalent cations. *Xenopus laevis* oocytes were injected with cRNAs encoding wild-type human polycystin-2 (polycystin-2, wild-type) and a human polycystin-2 protein without a 34-amino acid motif extending from amino acids 787-820 which is responsible for its preferential location in the endoplasmic reticulum [polycystin-2, Δ(aa787-820)], respectively. (a, b) To distinguish polycystin-2 localized at the cell surface from intracellular polycystin-2, cell surface proteins were labeled with biotin. A Western blot from a representative experiment demonstrates the increased presence of the mutant polycystin-2 protein at the cell surface (a) whereas comparable levels of both proteins

Journal of Cell Science • Supplementary information

are present intracellularly (b). Control oocytes without injection of the respective expression plasmids (control) were used to demonstrate the specificity of the signal detected with the anti-polycystin-2 antibody (for clarity, superfluous lanes on the same membrane were removed in order to position the control lane next to the lanes with the polycystin-2 signal). To validate separation of cell surface proteins from intracellular proteins, blots were re-probed using a rabbit anti-β-actin antibody. One representative blot is shown, quantitative densitometric data from four experiments are presented next to the respective Western blots together with the mean ± standard errors. The numbers on the left of the blots give the size of the molecular weight standard in kDa. *, $p < 0.05$; n.s., not significant. (c-e) Whole-cell currents were measured in control oocytes, oocytes expressing wild-type polycystin-2 (polycystin-2, wild-type) and polycystin-2 without the 34-amino acid responsible for its preferential location in the endoplasmic reticulum [polycystin-2, Δ(aa 787-820)]. Polycystin-2-mediated inward currents carried by Na^+ were elicited by removing divalent cations from the bath solution. Current measurements were performed and analyzed essentially as described in Fig. 2a, b. Summary *I/V* plots with mean values ± standard errors are shown in the left panels. Arrows indicate inward current values measured at -120 mV [$I_{(-120\ mV)}$]. Measurements from individual oocytes are summarized in the right panels and demonstrate significant polycystin-2-mediated inward currents in oocytes expressing polycystin-2, wild-type (d) and polycystin-2, Δ(aa787-820) (e), but not in control oocytes (c). Average *I/V* plots and $I_{(-120\ mV)}$ values represent results from 18 oocytes and 3 different oocyte preparations for each group. ***, $p < 0.001$; n.s., not significant.

Journal of Cell Science • Supplementary information

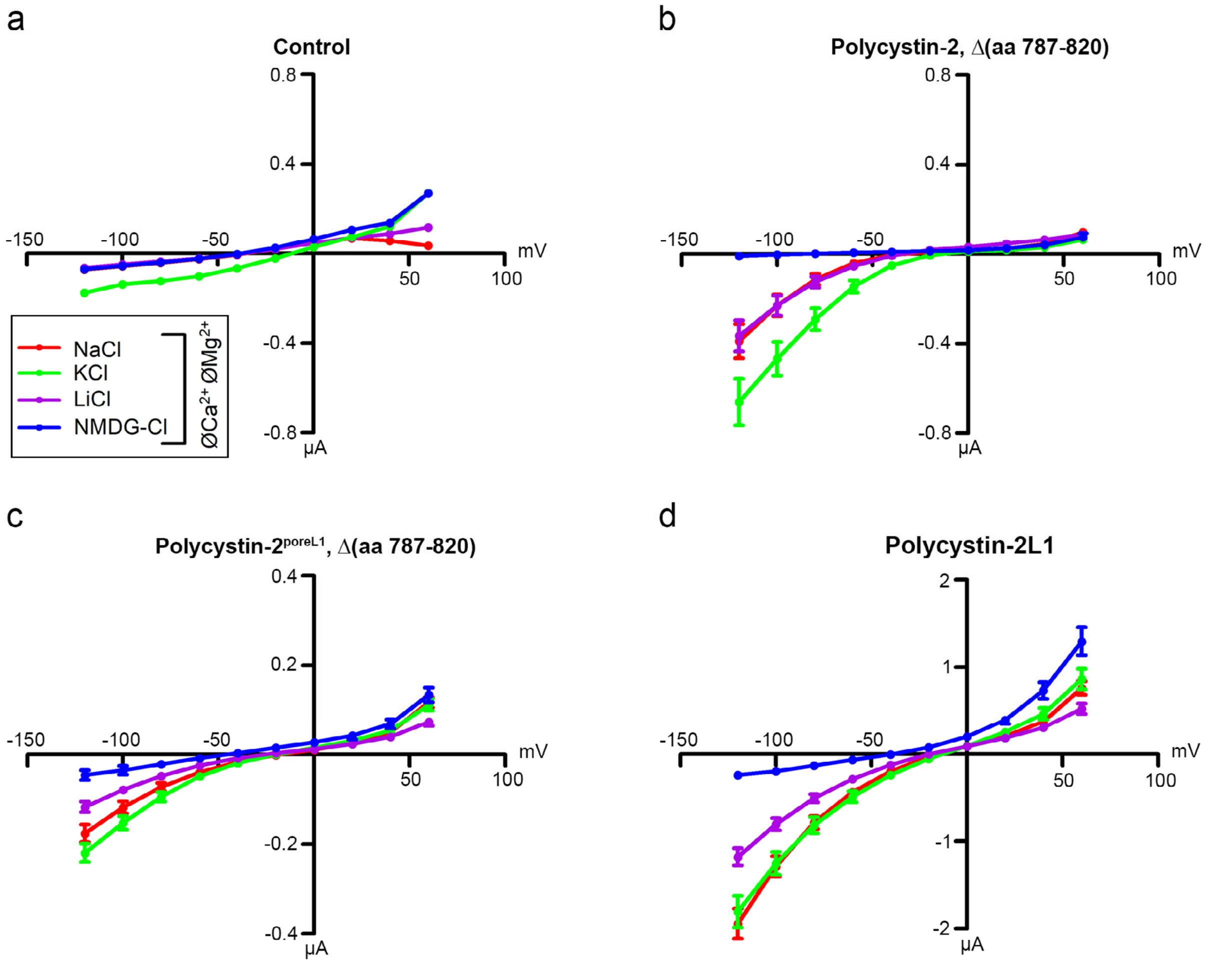

Fig. S2. Selectivity of polycystin-2, Δ(aa 787-820), polycystin-2^{poreL1}, Δ(aa 787-820), and polycystin-2L1 for Na$^+$, K$^+$ and Li$^+$ ions. Using a similar experimental design as shown in Fig. 2a and b, Na$^+$ was replaced in the bath solution by K$^+$, Li$^+$ and NMDG$^+$ in the absence of divalent cations and *I*/*V* plots were determined. To correct for endogenous oocyte currents (in particular K$^+$ currents), the average whole-cell current values measured in control oocytes (a) were subtracted from the corresponding individual whole-cell current values measured in oocytes from the same batch expressing polycystin-2, Δ(aa 787-820) (b), polycystin-2^{poreL1}, Δ (aa 787-820) (c) and polycystin-2L1 (d). In oocytes expressing polycystin-2, Δ(aa 787-820) we observed considerably higher inward currents in the presence of K$^+$ than in the presence of Na$^+$ and similar inward currents when Na$^+$ was replaced by Li$^+$. In contrast, in oocytes expressing polycystin-2L1 inward currents in the presence of K$^+$ were essentially identical to those in the presence of Na$^+$ with slightly smaller inward currents in

the presence of Li^+. Thus, polycystin-2, Δ(aa 787-820) conducts K^+ considerably better than Na^+ and Li^+ whereas polycystin-2L1 conducts K^+ and Na^+ equally well but has a slightly reduced conductance for Li^+. The inward currents in oocytes expressing polycystin-2^{poreL1}, Δ(aa 787-820) showed intermediate properties. Unlike the currents in oocytes expressing polycystin-2, Δ(aa 787-820), they were only slightly larger in the presence of K^+ than in the presence of Na^+. Moreover, like in oocytes expressing polycystin-2L1, they were slightly smaller in the presence of Li^+ than in the presence of Na^+. This suggests that polycystin-2^{poreL1} conducts Li^+ slightly less well than Na^+, with a marginally higher conductance for K^+ than for Na^+. Replacing Na^+ by K^+ and Li^+ caused corresponding reversal potential shifts which were used to estimate cation permeability ratios (Table S1). These are in good agreement with the qualitative conclusions reached from the inward current data. Results represent experiments from 23-31 oocytes and 4 different oocyte preparations, shown are the mean values ± standard errors.

Journal of Cell Science • Supplementary information

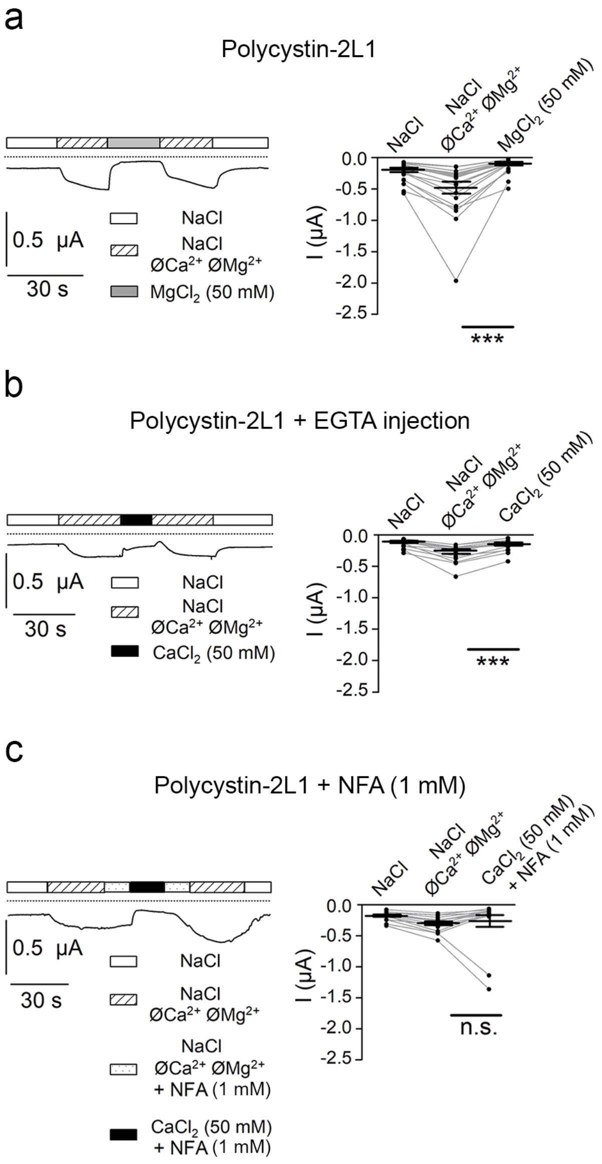

Fig. S3. The transient inward current in oocytes expressing polycystin-2L1 exposed to 50 mM $CaCl_2$ is not mimicked by 50 mM $MgCl_2$, can be prevented by buffering intracellular Ca^{2+} and by a chloride channel inhibitor. *Xenopus laevis* oocytes were injected with cRNA encoding polycystin-2L1, and removal of Ca^{2+} and Mg^{2+} from the bath solution was used to stimulate inward currents. (a) Subsequent application of 50 mM $MgCl_2$ reduced the inward currents (20 oocytes from 2 different oocyte preparations) as opposed to the transient inward current response observed with 50 mM $CaCl_2$ (see Fig. 2c). (b, c) Exposure to 50 mM $CaCl_2$ failed to elicit a transient inward current response in oocytes expressing polycystin-2L1 when oocytes were injected beforehand with 50 nl of 50 mM EGTA-K^+ (15 oocytes from 1 oocyte preparation) (b) and when 50 mM $CaCl_2$ was applied in

Journal of Cell Science • Supplementary information

the presence of the chloride channel inhibitor niflumic acid (NFA, 1 mM; 16 oocytes from 1 oocyte preparation) (c). The dotted line indicates zero current levels. Measurements from individual oocytes and mean values ± standard errors are shown. ***, $p < 0.001$.

Journal of Cell Science • Supplementary information

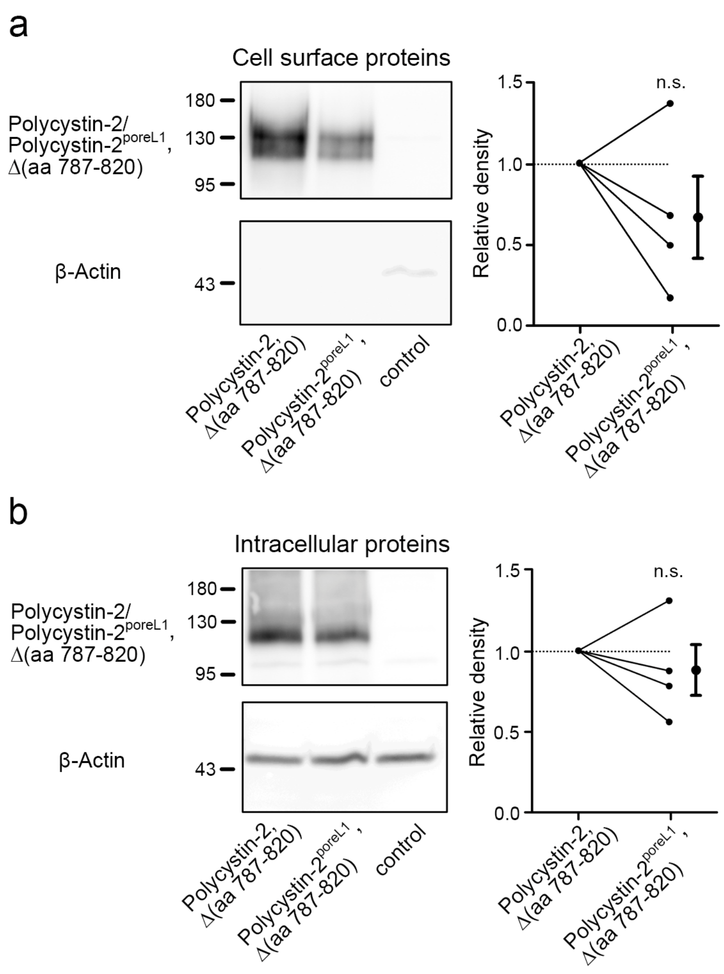

Fig. S4. Expression of polycystin-2^{poreL1}, Δ(aa 787-820) at the cell surface. *Xenopus laevis* oocytes were injected with cRNAs encoding human polycystin-2, Δ(aa 787-820) and polycystin-2^{poreL1}, Δ(aa 787-820), respectively. Control oocytes were used to confirm the specificity of the signal detected with an anti-polycystin-2 antibody in oocytes expressing polycystin-2, Δ(aa 787-820) and polycystin-2^{poreL1}, Δ(aa 787-820). Heterologous expression of polycystin-2, Δ(aa 787-820) and polycystin-2^{poreL1}, Δ(aa 787-820) at the cell surface (a) and intracellularly (b) was analyzed essentially as described in Fig. S1. Representative data from four independent experiments are shown together with summary data (mean ± standard error). The numbers on the left of the blots give the size of the molecular weight standard in kDa. n.s., not significant.

Journal of Cell Science • Supplementary information

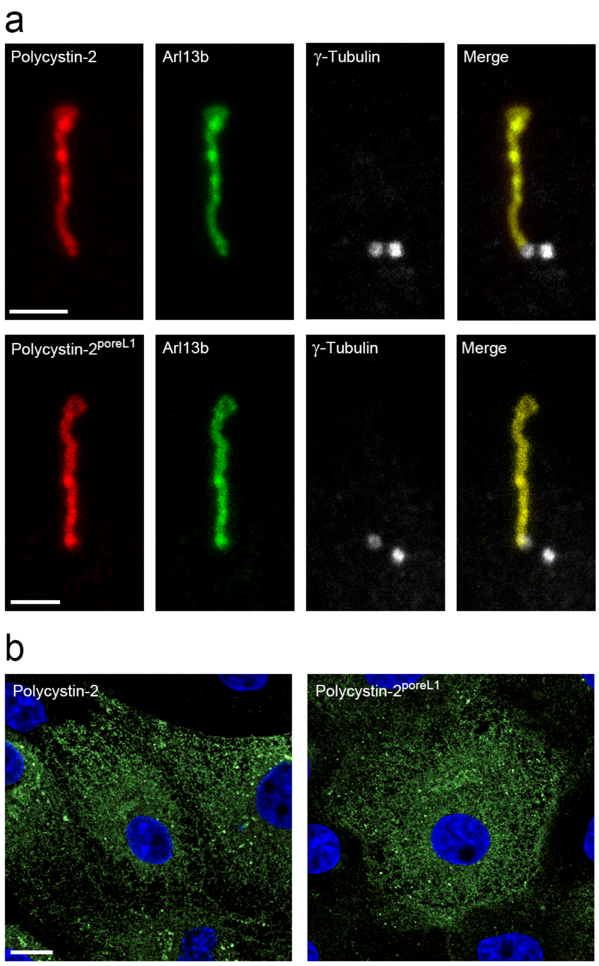

Fig. S5. Intracellular distribution of polycystin-2 and polycystin-2^{poreL1}. LLC-PK$_1$ cells were stably transfected with expression plasmids for the HA-epitope-tagged polycystin-2 and polycystin-2^{poreL1} proteins, respectively. Selected clones were stained with an antibody against the HA-epitope. (a) Costaining with an antibody against Arl13b marks the ciliary axoneme and with an antibody against γ-tubulin marks the basal body and the daughter centriole, thus demonstrating the presence of both proteins in the primary cilium. Bars, 2 µm. (b) The reticular pattern in the cytoplasm is indicative of the endoplasmic reticulum. Bar, 10 µm.

Journal of Cell Science • Supplementary information

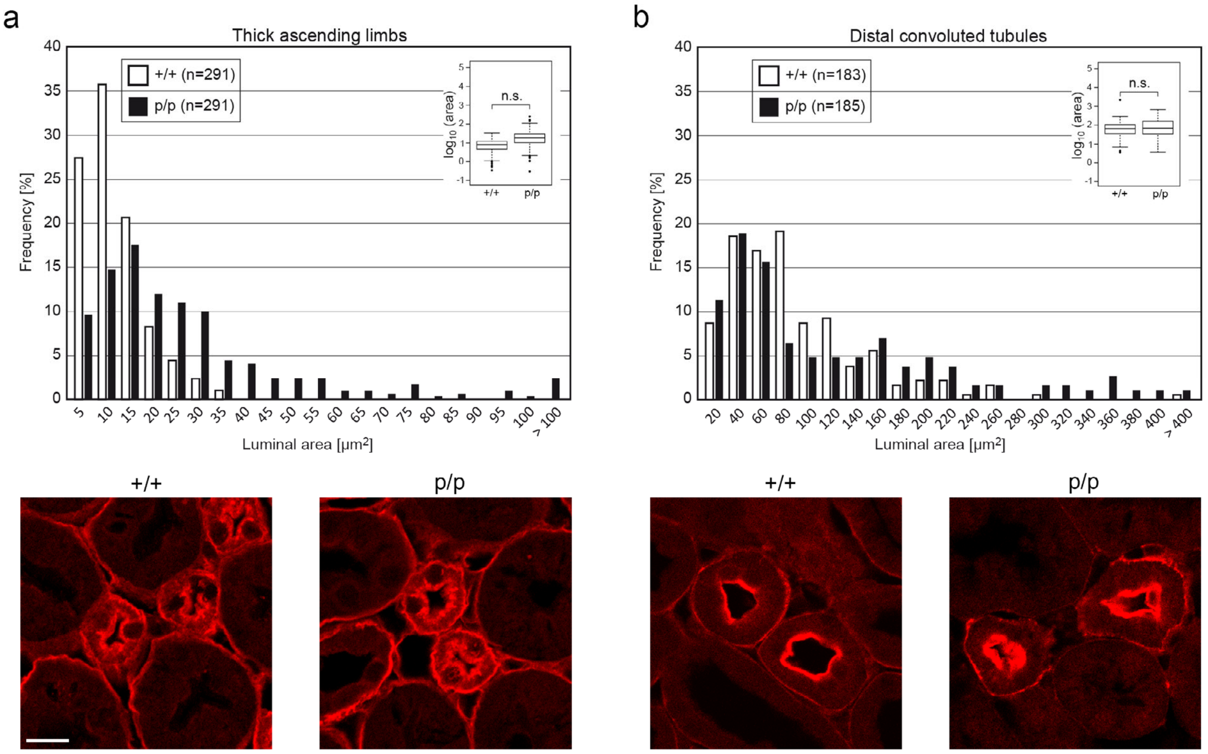

Fig. S6. Luminal areas of cortical thick ascending limbs and distal convoluted tubules in *Pkd2*$^{\mathrm{poreL1}}$ knock-in mice. Luminal areas of either nephron segment were not different between *Pkd2*$^{\mathrm{poreL1/poreL1}}$ knock-in (p/p) and wild-type (+/+) mice. Six-month old 129/Sv mice were used for analysis (4 wild-type and 6 homozygous *Pkd2^{poreL1}* knock-in mice). Boxes range from the 25th to the 75th percentile, the horizontal line in the box indicates the median, whiskers extend to data within 1.5 times inter-quartile range. n.s., not significant. Fluorescence images below the graphs show representative pictures of cortical thick ascending limbs (stained for uromodulin) and of distal convoluted tubules (stained for the Na^+/Cl^- cotransporter). Please note that the sections were also stained with an anti-laminin antibody. Bar, 20 µm.

Journal of Cell Science • Supplementary information

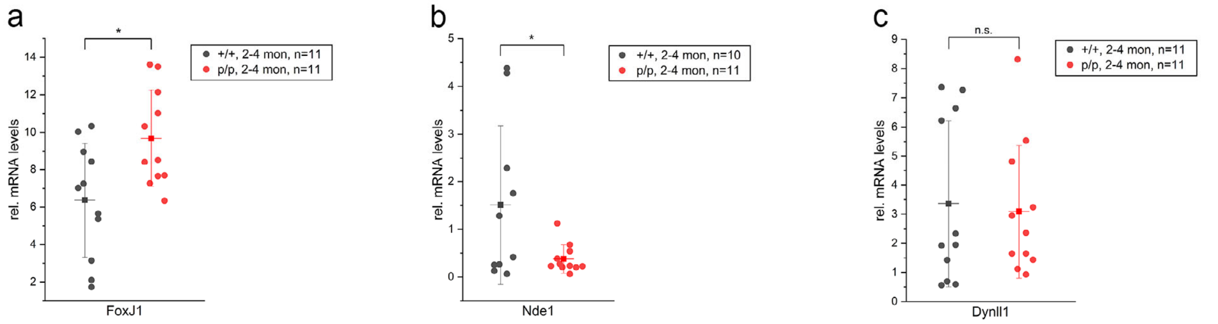

Fig. S7. Foxj1, Nde1 and Dynll1 mRNA levels in *Pkd2*$^{poreL1/poreL1}$ knock-in mice. Quantitative PCR analysis of total RNA isolated from collecting ducts shows higher Foxj1 and lower Nde1 mRNA levels in *Pkd2*$^{poreL1/poreL1}$ than in *Pkd2*$^{+/+}$ mice, whereas the mRNA levels for Dynll1 did not change. Approximately 150 to 200 collecting ducts each were harvested from 11 mice at an age of 2 to 4 months. Shown are the mean values ± standard deviations. *, $p < 0.05$; n.s., not significant.

Table S1. Effect of replacing Na^+ in the bath solution by K^+ and by Li^+ on average reversal potentials in *Xenopus laevis* oocytes expressing polycystin-2, Δ(aa 787-820), polycystin-2^{poreL1}, Δ(aa 787-820) and polycystin-2L1. Reversal potentials observed in the presence of Na^+ ($E_{rev,Na}{}^+$), K^+ ($E_{rev,K}{}^+$) and Li^+ ($E_{rev,Li}{}^+$) were estimated from the averaged *I*/*V*-curves shown in Fig. S2. Reversal potential shifts were calculated by subtracting $E_{rev,Na}{}^+$ from $E_{rev,K}{}^+$ ($\Delta E_{rev,\ K^+\text{-}Na^+}$) and from $E_{rev,Li}{}^+$ ($\Delta E_{rev,\ Li^+\text{-}Na^+}$) and were used to estimate permeability ratios (P_{Na}:P_K:P_{Li}). It should be mentioned that currents in oocytes expressing polycystin-2, Δ(aa 787-820) and polycystin-2^{poreL1}, Δ(aa 787-820) were much smaller than those in oocytes expressing polycystin-2L1, possibly due to a higher channel density at the cell surface. Thus, the relative contribution of overexpressed polycystin-2L1 to the overall whole-cell current including endogenous currents was larger than that of polycystin-2 and polycystin-2^{porL1}. As stated above (legend to Fig. S2) we corrected for endogenous oocyte currents by subtracting average whole-cell current values measured in corresponding control oocytes. Despite this correction the reversal potential measurements and the permeability ratios estimated from reversal potential shifts have to be interpreted with some caution.

	Polycystin-2, Δ(aa 787-820)	Polycystin-2^{poreL1}, Δ(aa 787-820)	Polycystin-2L1
$E_{rev,Na}{}^+$ [mV]	-36.8	-18.0	-15.2
$E_{rev,K}{}^+$ [mV]	-15.5	-20.1	-12.7
$E_{rev,Li}{}^+$ [mV]	-36.3	-26.2	-19.5
$\Delta E_{rev,\ K^+\text{-}Na^+}$ [mV]	21.3	-2.1	2.5
$\Delta E_{rev,\ Li^+\text{-}Na^+}$ [mV]	0.5	-8.2	-4.3
P_{Na} : P_K : P_{Li}	~1.0 : 2.3 : 1.0	~1.0 : 0.9 : 0.7	~1.0 : 1.1 : 0.8

Journal of Cell Science • Supplementary information

Table S2. Pore radii along the ion translocation axis. The distances between the constricting atoms in the selectivity filter of the pore region of polycystin-2 and the ion translocation axis were measured in the cryo-electron microscopical structure of polycystin-2 in its multi-ion state (PDB ID: 5mkf) (Wilkes et al., 2017). Corresponding distances were measured at the homologous positions in the cryo-electron microscopical structure of polycystin-2L1 (PDB ID: 5z1w) (Su et al., 2018). The radii for polycystin-2^{poreL1} were derived from the structural homology model (this work). In the case of the most proximal amino acid (D643 in polycystin-2 and polycystin-2^{poreL1}, D523 in polycystin-2L1) the carbon atom immediately adjacent to the carboxyl group was taken as a reference point, in the case of the two following amino acids the oxygen atom of the carbonyl group was taken.

Polycystin-2		Polycystin-2^{poreL1}		Polycystin-2L1	
Amino acid	Radius [Å]	Amino acid	Radius [Å]	Amino acid	Radius [Å]
D643	5.4	D643	7.5	D523	6.1
G642	3.8	G642	5.3	G522	3.6
L641	2.9	L641	4.4	L521	5.0

Journal of Cell Science • Supplementary information

Table S3. Thermodynamic and physical characteristics of relevant cations. Data were taken from (Tansel, 2012). n.a., not available.

Cation	Ionic radius [Å]	Hydrated radius [Å]	Hydration free energy [-kJ/mol]	Entropy of hydration [kj/mol]
Li^+	0.76-0.78	3.40-3.82	515-544	n.a.
Na^+	0.98-1.16	2.77-3.60	365-435	-5
K^+	1.33-1.52	2.01-3.31	271-351	+34
Ca^{2+}	1.00-1.23	4.12-4.20	1,306-1,650	-174

Journal of Cell Science • Supplementary information

References

Su, Q., Hu, F., Liu, Y., Ge, X., Mei, C., Yu, S., Shen, A., Zhou, Q., Yan, C., Lei, J. et al. (2018). Cryo-EM structure of the polycystic kidney disease-like channel PKD2L1. *Nat. Commun.* **9**, 1192.

Tansel, B. (2012). Significance of thermodynamic and physical characteristics on permeation of ions during membrane separation: Hydrated radius, hydration free energy and viscous effects. *Sep. Purif. Technol.* **86**, 119-126.

Wilkes, M., Madej, M. G., Kreuter, L., Rhinow, D., Heinz, V., De Sanctis, S., Ruppel, S., Richter, R. M., Joos, F., Grieben, M. et al. (2017). Molecular insights into lipid-assisted Ca^{2+} regulation of the TRP channel polycystin-2. *Nat. Struct. Mol. Biol.* **24**, 123-130.

Journal of Cell Science • Supplementary information
